# The Effect of Locomotion Mode on Body Shape Evolution in Teleost Fishes

**DOI:** 10.1093/iob/obab016

**Published:** 2021-05-18

**Authors:** Sarah T Friedman, Samantha A Price, Peter C Wainwright

**Affiliations:** 1 Department of Evolution and Ecology, University of California Davis, Davis, CA 95616-5270, USA; 2 Department of Biological Sciences, Clemson University, Clemson, SC 29634, USA

## Abstract

Teleost fishes vary in their reliance on median and paired fins (MPF) or undulation of the body (BCF) to generate thrust during straight-line, steady swimming. Previous work indicates that swimming mode is associated with different body shapes, though this has never been empirically demonstrated across the diversity of fishes. As the body does not play as active a mechanical role in steady swimming by MPF swimmers, this may relax constraints and spur higher rates of body shape diversification. We test these predictions by measuring the impact of the dominant steady swimming mode on the evolution of body shape across 2295 marine teleost fishes. Aligning with historical expectations, BCF swimmers exhibit a more elongate, slender body shape, while MPF propulsion is associated with deeper and wider body shapes. However, in contrast to expectations, we find that BCF propulsion is associated with higher morphological diversity and greater variance around trait optima. This surprising result is consistent with the interpretation that stronger functional trade-offs stimulate phenotypic evolution, rather than constrain it.

## Introduction

Multifunctionality is among the most general features of multicellular organisms. Many body parts perform multiple functions in an organism, setting up conflicts that may result in structural compromises that do not maximize any single function. The ubiquity of multifunctionality in whole organisms suggests that design trade-offs between functions may be one of the dominant factors constraining phenotypic evolution (Arnold 1992; Ghalambor et al. 2004; Salathé et al. 2006; Pritykin et al. 2015). However, studies have also demonstrated that trade-offs can stimulate diversity, particularly in biomechanical systems (Holzman et al. 2012; Muñoz et al. 2017, 2018). Given these conflicting findings, it is unclear what the long-term consequences of multifunctional trade-offs are on the macroevolutionary diversity of phenotypes.

In the present study we explore this issue by leveraging the relationship between swimming mode and body shape in fishes. Across fishes, there is a major distinction between species that locomote by undulating the body and caudal fin (BCF) and those that propel themselves with movements of the median and paired fins (MPFs) (Webb 1984a; Sfakiotakis et al. 1999). While most species are thought to undergo gait transitions from MPF to BCF swimming as they increase speed (Drucker and Jensen 1996; Cannas et al. 2006; Feilich 2017; George and Westneat 2019), the mode that is dominant during steady, straight-line swimming tends to be distinct and varies across taxa (Webb 1984a, 1984b; Korsmeyer et al. 2002; Hale et al. 2006). The mechanical role of the body differs substantially between these swimming modes as the lateral body surface and caudal fin only generate thrust during steady swimming for fishes using BCF (Webb 1984a, 1984b; Sfakiotakis et al. 1999; Blake 2004). Since the body is uninvolved in thrust production during MPF swimming, species that make more extensive use of this mode may experience fewer hydrodynamic demands on body form permitting greater diversity in body shape (Blake 2004). Swimming performance and mode are thought to be under intense selective pressure in fishes (Tytell et al. 2010) and likely affect body shape adaptation (Langerhans and Reznick 2010), making MPF swimmers a candidate system with a relaxation of constraints associated with the shift in swimming mechanism.

The difference in the mechanical role that the body plays during BCF and MPF swimming suggests that species with different modes may also exhibit different body shapes. This distinction is seen clearest at the extremes of performance where it has long been recognized that species with very high BCF cruising performance usually have a narrow caudal peduncle, lunate caudal fin, and a streamlined body shape (Webb 1984b. In contrast, MPF locomotion is thought to perform best in slow swimming, maneuverable species that are often characterized by a short, deep, laterally compressed body (Blake 1983; Korsmeyer et al. 2002). This body shape provides instability and positions fin surfaces at high-leverage positions with respect to the fish center of mass, both of which contribute to sharp, precise turning motions (Blake 1983, 2004); Webb 1984a, 1984b; Korsmeyer et al. 2002). Classical examples of BCF cruising specialists include scombrids and carangids, while butterflyfishes and surfperches are considered MPF maneuvering specialists (Webb 1984b; Blake 2004).

While performance extremes may be associated with distinct phenotypes, fish body shapes are known to be extremely diverse (Collar et al. 2013; Claverie and Wainwright 2014). As the relationship between dominant swimming mode and body shape has never been surveyed across the diversity of teleost fishes, it is not even known whether a general correlation exists. For example, species that emphasize the use of MPF swimming exhibit a striking diversity of body shapes, including cuboid boxfish, deep-bodied surgeonfish, and sigmoidal seahorses. Indeed, the principal energetic advantages of MPF locomotion—efficiency, precision, and maneuverability during slow speed swimming—can be achieved with a variety of body forms (Korsmeyer et al. 2002; Blake 2004) and functional properties (Ghalambor et al. 2003; Webb and Weihs 2015). Swimming modes are also not fixed within a species (Ellerby and Gerry 2011). Most species employ MPF swimming at very low speeds and undergo a gait change to BCF at some intermediate speed (Drucker and Jensen 1996; Webb 1998). Nevertheless, species differ in the speed at which they undergo this gait change (Feilich 2016) so that a dominant swimming mode during cruising can usually be identified for a species. Fish bodies also evolve to simultaneously adapt to additional functions, including feeding, habitat, and structural defense against predators, all of which can have significant effects on body shape evolution (Price et al. 2015; Friedman et al. 2016, 2020). This diversity in both locomotion mode and selective pressures is worth emphasizing because it suggests there is a complex relationship between body shape and swimming mode across fishes.

We analyze the impact of swimming mode on body shape evolution in teleost fishes using a data set of body shape measurements from 2295 species. To explore the dynamics of morphological evolution, we estimate the optimal body shape and the variance of traits around the optima (the combined effects of selection and rate of evolution) for BCF and MPF swimmers using evolutionary model fitting. Our expectation is that BCF swimmers have a more slender body, intermediate body depth, and narrow caudal peduncle. We expect MPF swimmers will be deeper bodied, short, and laterally compressed, as previous researchers and studies have suggested (Webb 1984a, 1984b; Blake 2004; Larouche et al. 2020). Given the possibility that the central role of the body in generating thrust during steady BCF swimming places stabilizing selection on body shape, we predict that the variance around trait optima will be lower in BCF swimmers. Because the body is not used to propel the fish during MPF swimming, these species may experience fewer constraints on body form evolution, resulting in greater diversity and higher variance of traits in this group (Blake 2004; Langerhans and Reznick 2010).

## Materials and methods

### Data collection and preparation

This study made use of an existing large data set on body shapes of teleost fishes (Price et al. 2019). Briefly, these data were collected from specimens from the Smithsonian National Museum of Natural History using hand-held linear measurements to capture major body dimensions from three adult specimens across over 6100 teleost species. We initially trimmed this data set to 3344 marine species and then removed all benthic fishes (identified from Friedman et al. 2020) to focus our analyses on species that occupy demersal and pelagic realms. For each of the 2295 species in our study we obtained the species averages for six body dimensions: standard length, maximum body depth, maximum body width, minimum caudal peduncle depth, minimum caudal peduncle width, and head depth. We also analyzed lower jaw length and mouth width to explore the association between locomotion mode and the evolution of feeding traits (Keast and Webb 1966; Larouche et al. 2020). For further details on data collection, measurements, and methods see Price et al. (2019).

Prior to analysis, linear traits were ln-transformed and size corrected by taking the residuals of a phylogenetic regression on body size using the R package phytools (Revell 2012). We used a time-calibrated phylogeny of ray-finned fishes pruned to our species list for all comparative analyses (Rabosky et al. 2018). Here, body size was a composite metric calculated as the geometric mean of the three major body shape dimensions: standard length, maximum body depth, and maximum body width (Mosimann 1970; Klingenberg 2016). All analyses for this study were implemented in the R statistical computing environment version 3.6.2 (R Core Team 2017).

Species were categorized into two broad locomotion mode categories: MPF and BCF based on their dominant swimming mode during straight-line, steady cruising. We acquired swimming mode data through comprehensive literature searches of swimming behavior, evaluation of online videos, and personal observations of fish locomotion both in aquaria and *in situ* (Supplementary Table S1). Species were categorized as MPF swimmers if they primarily propelled themselves by oscillating and/or undulating their median (dorsal/anal) and/or pectoral fins, while species were categorized as BCF if they swam by undulating their body and caudal fin. We note that the majority of species use different swimming modes depending on the situation and many species transition from MPF to BCF with increasing swimming speed. We categorized species based on primary observations of steady swimming during an uninterrupted linear trajectory of at least several body lengths, as this would be the mode used by the fish during straight, sustained aerobic movement about the habitat. If both swimming modes were employed simultaneously or the categorization was otherwise ambiguous, we removed the species from the study.

### Morphological evolution

We conducted a principal components analysis (PCA) using the correlation matrix to visualize morphospace occupancy both across the dataset and with respect to swimming mode. Although phylogenetic PCA is a common technique used in macroevolutionary studies, here we are interested in the primary dimensions of morphological variation across all fishes, regardless of phylogenetic structuring. To determine the overall diversity of forms in each locomotion mode, we calculated multivariate morphological disparity (variance) across the eight morphological traits. We used both a phylogenetic MANOVA and a series of ANOVAs for the eight traits to determine if there were significant differences in average body shape between MPF and BCF swimmers. The ANOVAs and variance calculations were implemented with 1000 simulations using the geomorph package (Adams et al. 2019).

To determine if the dynamics of morphological evolution differ between MPF and BCF swimmers, we first reconstructed the evolution of locomotion mode across the phylogeny using stochastic character mapping (simmaps; root.station = FALSE), allowing for asymmetric transition rates between swimming modes, as this was the preferred model based on AIC comparisons (Revell 2012). For each of the eight morphological traits, we then used OUwie version 1.50 (Beaulieu and O’Meara 2015; root.station = FALSE) with 100 simmaps to compare five models—two Brownian motion and three Ornstein–Uhlenbeck (OU) models—which successively allowed for different combinations of evolutionary parameters to vary with locomotion mode. Single rate Brownian motion (BM1) does not allow for the rate of evolution (*σ*^2^) to vary with locomotion mode, while the multi-rate Brownian motion model (BMS) allows for different rates in MPF and BCF swimmers. OU models incorporate an additional parameter, *θ*, which is generally interpreted as an adaptive optimum. We implemented three OU models: a single peak OU model (OU1), a multi-peak OU model (OUM), and a multi-peak multi-rate OU model (OUMV). Though there is a third parameter that OU models can accommodate, *α*, which is commonly interpreted as a selective pull toward the morphological optimum, we chose not to incorporate models that allow this parameter to vary with regime. It has been shown that the similar effects of alpha and sigma parameters in OU models can lead to model non-identifiability, as well as inaccurate estimates of alpha (Si Tung Ho et al. 2014; Cooper et al. 2016). We therefore decided to omit such models from this study to reduce ambiguity in parameter estimates. However, removing these models necessitated that any differences in variance around the optimum were attributed to *σ*^2^. Thus, estimates of rate in this study are more accurately a measure of variance around the trait optimum, as we cannot disentangle the effects of *σ*^2^ and *α*. To summarize the relative influence of stochastic factors in the adaptive process, we calculated the stationary variance (*σ*^2^/2*α*) of the joint OU–BM process (Hansen 1997).

We checked the results of the OUwie analyses for positive eigenvalues, which indicate reliable estimates (Beaulieu et al. 2012). Model fit was evaluated using a modified Akaike information criterion (AICc), which converges to AIC when samples are large. To determine if we have the power to distinguish between the models, we also simulated a dataset under the best fit parameters for the OUMV model across the phylogeny using the function *OUwie.sim*. We then recursively ran our model-fitting framework with 100 simmaps to establish if we could recover the original model and parameters, thereby demonstrating statistical power.

## Results

### Morphological evolution

The first two PC axes accounted for 65.3% of morphological variation across the dataset (Supplementary Table S2). Principal component one (PC1) reflected body elongation as it involved a concordant increase in standard length and decrease in body depth. The morphospace shows strong phylogenetic partitioning, with eels retaining high values along PC1, while lower values tend to be occupied by deep bodied reef dwelling fishes, such as surgeonfish and filefish (Fig. 2). PC2 is driven by differences in body width, with robust diodontids scoring high on PC2, contrasted with narrow-bodied fishes scoring low on PC2. Swimming mode exhibits patterning in morphospace, with MPF swimmers primarily occupying the left of the morphospace, corresponding with deeper body shapes, and BCF swimmers dominating the right with elongate body shapes (Fig. 2). The morphospace also reveals that, while morphological diversity is primarily distributed along PC1 for BCF swimmers, fishes that use MPF propulsion tend to vary more along PC2. This suggests that species that emphasize different swimming modes may primarily diversify along different morphological axes.

**Fig. 1:**
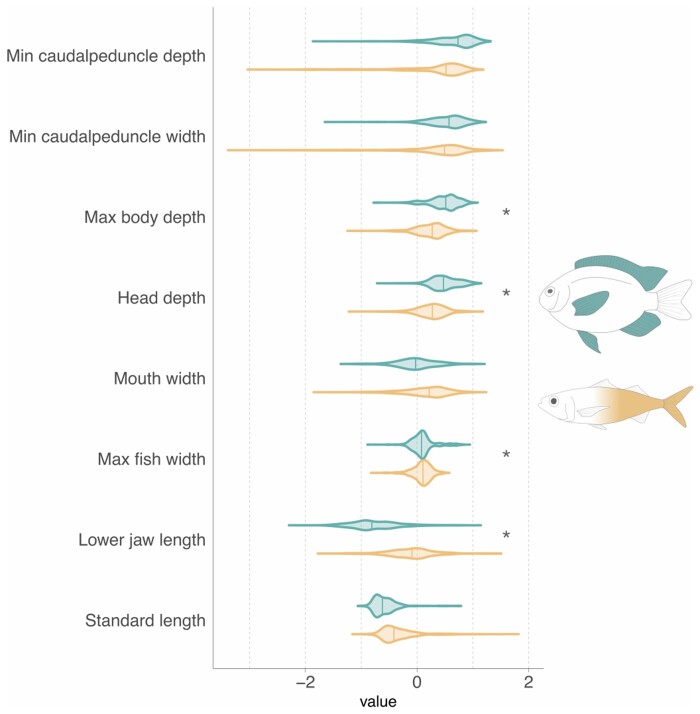
Distribution of all eight traits by locomotion mode. Asterisks indicate the trait was found to be statistically significant in the phylogenetic ANOVA. Fish drawings to the right represent the species closest to the median body shape for each swimming mode (BCF: *Erythrocles monodi*; MPF: *Neoglyphidodon polyacanthus*).

**Fig. 2:**
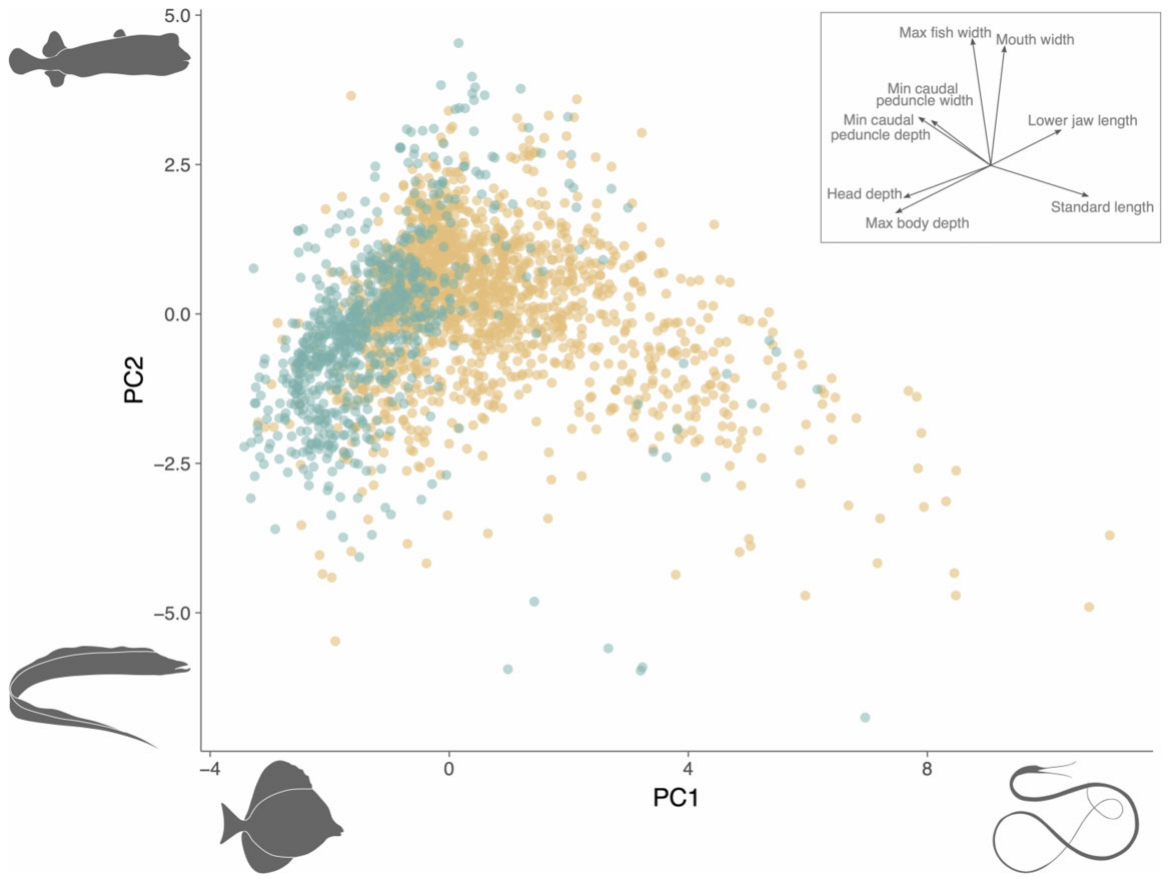
Morphospace of the eight traits describing fish body shape with inset arrows to visualize the loadings on the first two PCs. Each point is a single species colored by locomotion mode (BCF: yellow, MPF: blue) and silhouettes illustrate the extreme shapes along each axis (PC1_max_: *Nemichthys curvirostris*; PC1_min_: *Zebrasoma scopas*; PC2_max_: *Chilomycterus antillarum*; PC2_min_: *Trichiurus lepturus*).

BCF swimmers exhibit 1.87 times the trait variance of MPF fishes (BCF = 1.49, MPF = 0.795, *P* < 0.05), although body shape is highly variable within each mode (Figs. 1 and 2). Our phylogenetic MANOVA showed a significant difference in average body shape between BCF and MPF swimmers (*P* = 0.01, *F* = 4.10, *Z* = 2.18). The individual phylogenetic ANOVAs revealed that this result is primarily driven by significant differences in maximum body depth, head depth, lower jaw length, and maximum body width (Fig. 1). The average MPF swimmer has a deeper body and head, while the mean BCF swimmer is more laterally compressed and has longer jaws.

**Fig. 3:**
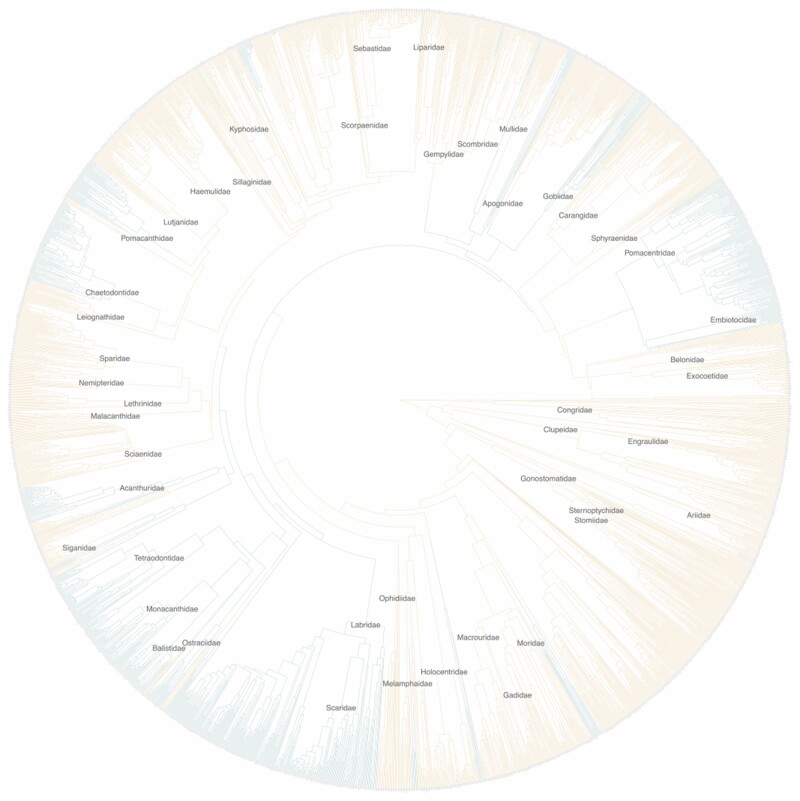
Representative stochastic character reconstruction showing locomotion mode transitions across the phylogeny (BCF: yellow; MPF: blue). Names of families with more than 15 species present are printed at the node corresponding to the most recent common ancestor.

Across the 100 stochastic character maps, we find an average of 40.58 transitions between locomotion modes. Of these transitions, 23.01 are from MPF to BCF swimming, indicating that there are roughly equivalent transition rates for both directions of locomotion mode transitions. The root node was constructed as BCF in 100% of the stochastic character reconstructions, indicating a high probability that it is the ancestral swimming mode of teleost fishes (Fig. 3).

**Fig. 4:**
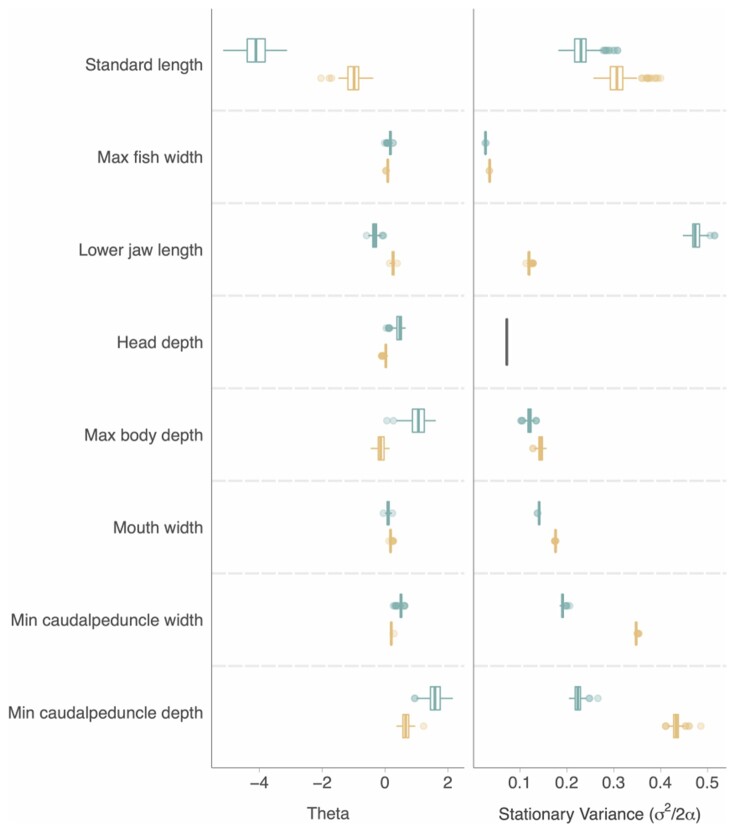
Parameter results for the best fit model from the OUwie analysis for each trait and regime (BCF: yellow; MPF: blue). All traits are best fit by an OUMV model, with different theta and sigma values for each locomotion mode, except head depth, which is best fit by an OUM model, with a single sigma value for both regimes. All sigma estimates are multiplied by 100 for ease of interpretation.

Our OUwie analyses of the morphological traits revealed a best fit model of OUMV for all traits (100/100 simmaps) except head depth, which had a best fit model of OUM (98/100 simmaps). In other words, all traits have different optima for MPF and BCF swimmers and all traits except head depth have different amounts of variance around the optima for the two swimming modes (Fig. 4 and Supplementary Table S3). BCF swimmers have higher stationary variance in all traits except lower jaw length. The greatest differences in stationary variance between the two modes are in the caudal peduncle measurements (1.8–1.9× higher in BCF swimmers). Across all morphological traits, BCF swimmers have 1.27× greater stationary variance than MPF swimmers, on average. The largest differences in the morphological optima between the locomotion modes are in standard length and maximum body depth, such that BCF swimmers are evolving toward a more elongate, less deep body shape than MPF fishes (Fig. 4). The optima of MPF swimmers characterize fish with a deeper body, a thicker caudal peduncle, and shorter jaws, consistent with our predictions.

The simulations under our best fit model (OUMV) indicated that we have substantial power to distinguish between all five evolutionary models, recovering the original model in all 100 simmaps (Supplementary Fig. S1). The AICc estimates show virtually no overlap and clearly favor models that incorporate rate variation over those that do not. We also have decent statistical power to recover the empirical parameter estimates that the data were simulated under (Supplementary Fig. S1).

## Discussion

The expectation that swimming mode has a substantial effect on body shape has been central to interpretations of fish diversity for at least the past century (Hoerner 1965; Lighthill 1969; Webb 1984a, 1984b; Videler 1993; Triantafyllou et al. 2000). Our survey of 2295 species of marine fishes returned strong support for some expectations from this literature but raises questions about others. While we find BCF swimming is the ancestral and most common swimming mode in marine teleosts, we reconstruct about 23 transitions to MPF swimming and a roughly equal rate of transitions back to BCF swimming, indicating that teleost lineages have periodically responded to ecological challenges by altering locomotor mode. As predicted, we find a significant difference in the average body shape of BCF and MPF swimmers. The average MPF swimmer is deeper-bodied, shorter, and more laterally compressed with shorter jaws, while BCF swimmers are more elongate, and shallower-bodied, with a narrower caudal peduncle. However, body shape diversity is both extremely high within each mode and broadly overlapping between modes. The effect of swimming mode on body shape is not a rule but a significant trend with many exceptions. Lastly, in striking contrast to our expectations, BCF fishes exhibit 1.8 times as much body shape diversity as MPF species and greater stationary variance in all locomotion-related traits.

In spite of considerable variation among species, the differences in body shape between MPF and BCF swimmers generally match expectations. The short, deep, laterally compressed body shape that we find is common in MPF species is thought to confer efficiency at slow speeds and increased maneuverability (Webb 1984b; Blake 2004). A short body with large lateral surface area allows for a tighter turning radius (Webb 1982; Domenici and Blake 1997), suggesting that this average body shape and MPF swimming are well-suited for structurally-complex habitats. Indeed, we find that many of the clades that are known for diversification on reefs: wrasses, damselfishes, and butterflyfishes, are primarily or entirely composed of MPF swimmers. A deeper caudal peduncle would also enhance acceleration during fast starts by MPF swimmers (Webb 1982, 1984b). Interestingly, the clades that most strongly contribute to morphological variation within this mode—pufferfishes and filefishes—primarily vary in body width, suggesting that alterations along this axis may not functionally impede MPF locomotion. As predicted, we find that BCF swimmers have a more streamlined body shape and a narrow caudal peduncle, features that simultaneously reduce drag and increase thrust during steady BCF swimming (Webb 1982, 1984b; Blake 2004). In our dataset, BCF swimmers are composed of eels, jack, and, tuna and their allies, among many other lineages. Much of the body shape diversity in this mode results from changes to elongation (standard length and body depth) and deep-sea clades like dragonfishes, hatchetfishes, and bristlemouths contribute disproportionately to this variation. Studies have demonstrated that the deep-sea fosters rapid morphological evolution (Martinez et al. 2021), suggesting that BCF swimming (and the associated elongate body shape) may also serve as an adaptation for efficient slow locomotion. Though the relationship between locomotion mode and body shape has been extensively discussed (Webb 1984a, 1984b, 1988), this is the first broad-scale, quantitative confirmation of the relationship.

There are notable exceptions to the relationship between locomotion mode and average body shape described above. We find two clades of MPF swimmers with highly elongate bodies: ribbonfishes and pipefishes (and their allies). Furthermore, some of the most deep-bodied species in our dataset are BCF swimmers, including batfishes (Ephippidae), moonfish (Menidae), and deep sea hatchetfishes (Sternoptychidae). There are numerous aspects of swimming mechanics that we have not accounted for in our categorization system that likely contribute to variation within each mode. Median-paired fin swimming is a broad category that encompasses fishes that swim with any combination of their pectoral, dorsal, and anal fins. This variation in anterior–posterior placement of thrust generation may influence locomotor mechanics (Sfakiotakis et al. 1999) and the consequences of body shape. Similarly, from eels to tuna, BCF swimmers engage varying proportions of their body in propulsion, some relying more on the caudal fin to generate thrust (Lindsey 1978; Donley and Dickson 2000). Therefore, there are varying degrees of coupling between body shape and swimming mechanics contained within the BCF locomotion category. Body shape has also been shown to evolve in tandem with fin shape, such that different configurations can have a strong effect on locomotion performance and evolution (Feilich 2016; Larouche et al. 2017). The broad scope of this study limits our resolution, therefore future work would benefit from a more nuanced approach to classifying locomotion mechanics.

### Tempo of body shape evolution

We predicted that the decoupling of the lateral body surface from a mechanical role in generating thrust during steady locomotion may allow MPF swimmers to respond to the selective pressures on other functions of body shape (Langerhans and Reznick 2010), resulting in greater diversification of body shape (Blake 2004). However, we find that axial undulators (BCF swimmers) have 1.87 x the morphological variability and 1.27× higher stationary variance on average when compared with MPF species. We also note that, while BCF swimmers have higher stationary variance for most traits, the traits that are involved in feeding exhibit a different trend. MPF propulsion is associated with elevated stationary variance around the lower jaw length optimum (Fig. 4). Although BCF swimmers have higher stationary variance around the optimum for mouth width, the optimum itself (theta) differs little between MPF and BCF swimmers. These findings complement previous work on habitat-associated morphological evolution (Friedman et al. 2020) and suggest that functional traits relevant to feeding are under different selective pressures than those related to locomotion.

Our finding of higher stationary variance in BCF swimmers compliments a suite of studies that have demonstrated a pattern of rapid evolution in multi-functional traits (Holzman et al. 2012; Anderson and Patek 2015; Muñoz et al. 2017, 2018; Moen 2019). For example, it has been shown that the most mechanically sensitive link of a four-bar linkage system has the fastest rates of evolution in mantis shrimp and fish jaws (Muñoz et al. 2018). A study on suction feeding performance in multiple fish families also demonstrated that the traits associated with stronger functional trade-offs conferred faster rates of evolution (Holzman et al. 2012). These results imply that the multiple competing selective pressures imposed on complex, multi-functional traits may serve to flatten the adaptive landscape by providing multiple potential configurations with equivalent biomechanical solutions (Wainwright et al. 2005; Muñoz et al. 2018). Our data set shows pervasive many-to-one mapping of fish body shape to swimming mode as both MPF and BCF swimmers contain extensive variation in body shape. While this suggests the possibility of many-to-one mapping of body shape to swimming performance, confirmation of this possibility will have to await more detailed studies of the diversity of swimming performance in fishes. Furthermore, the underlying mechanism by which multi-functional traits may bias the tempo and mode of morphological evolution remains relatively unexplored and presents a fruitful area for future work.

## Conclusions

Although our categorization system glosses over much subtlety in swimming mechanics, we find strong evidence that locomotion mode has consequences for the evolution of fish diversity, supporting long-held predictions about the interplay between fish shape and swimming performance. Contrary to expectations, we also find that BCF locomotion is associated with higher stationary variance around trait optima and greater morphological diversity. The strong tradeoffs between adaptations for steady swimming and other ecological functions have resulted in greater body shape diversification in BCF species. Complex traits with strong performance tradeoffs, such as body shape, may either elevate rates of morphological evolution or reduce constraints, resulting in a decoupling between the evolution of form and function. Mounting evidence indicates that these patterns associated with multi-functional traits may be more of a general rule linking biomechanics and morphological diversification. We recommend that this framework be extended to other taxa and systems to better understand the role of multi-functional traits in biasing the tempo and mode of evolution.

## Supplementary Material

obab016_Supplementary_Data
