# Supplementary material for: The Effect of Locomotion Mode on Body Shape Evolution in Teleost Fishes
Source: Integr Org Biol. 2021 May 18;3(1):obab016. doi: 10.1093/iob/obab016 (PMC8341890; doi:10.1093/iob/obab016)
Supplement: obab016_Supplementary_Data [file obab016_supplementary_data.zip › SUPPLEMENTAL MATERIALS.pdf]

SUPPLEMENTAL MATERIALS

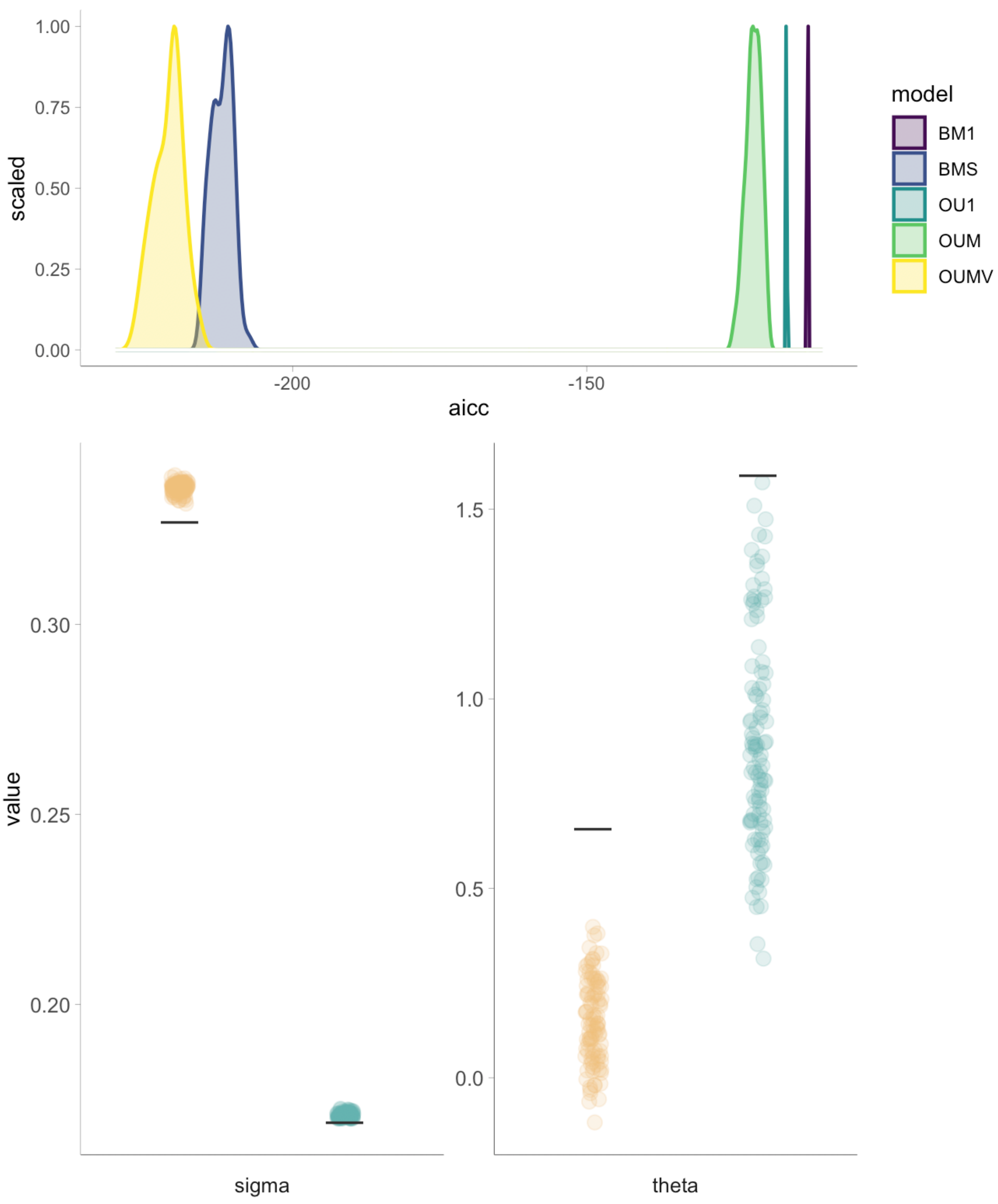

Figure S1. The top plot shows the distribution of AICc values for each model across 100 stochastic character maps (simmaps) for the data simulated under the OUMV model. The bottom plots show the reconstructed parameter values for the best fit model (OUMV) across 100 simmaps. Empirical parameter values are represented by the horizonal grey lines.

Table S2. Trait loadings on the first two components of the PCA on the entire dataset.

Trait	PC1	PC2
Standard length	0.487	-0.154
Max body depth	-0.474	-0.237
Max fish width	-0.093	0.633
Head depth	-0.433	-0.160
Lower jaw length	0.350	0.177
Mouth width	0.070	0.596
Min caudalpeduncle depth	-0.359	0.240
Min caudalpeduncle width	-0.295	0.223

Table S3. Comparisons of five evolutionary model fits for each linear trait (median values across 100 replications). Bolded rows represent the best fit model as determined by AICc.

Trait	Model	AICc	ΔAICc	lnL	σ_{BCF}	σ_{MPF}	$variance_{BCF}$	$variance_{MPF}$	θ_{BCF}	θ_{MPF}
Head depth	BM1	-2139	142.8	1071.5	0.001	-	-	-	-	-
	BMS	-2145.4	136.4	1075.7	0.001	0.001	-	-	-	-
	OU1	-2266.5	15.3	1137.3	0.001	-	0.078	-	927062.23	-
	**OUM**	**-2281.8**	**0**	1145.9	0.001	-	0.072	-	0.025	0.473
	OUMV	-2280	1.8	1146	0.001	0.001	0.072	0.073	0.024	0.47
Lower jaw length	BM1	1183.5	862	-589.7	0.004	-	-	-	-	-
	BMS	498.4	176.9	-246.2	0.002	0.01	-	-	-	-
	OU1	833.5	512	-412.7	0.006	-	0.19	-	-0.002	-
	OUM	766.7	445.3	-378.4	0.006	-	0.166	-	0.077	-0.588
	**OUMV**	**321.5**	**0**	-154.7	0.003	0.011	0.119	0.474	0.261	-0.327
Max body depth	BM1	-3451	19.3	1727.5	0.001	-	-	-	-	-
	BMS	-3464.1	6.1	1735.1	0.001	0.001	-	-	-	-
	OU1	-3455.7	14.6	1731.8	0.001	-	0.157	-	1464409.869	-
	OUM	-3465.7	4.6	1737.9	0.001	-	0.139	-	-0.157	1.074
	**OUMV**	**-3470.3**	**0**	1741.1	0.001	0.001	0.144	0.12	-0.14	1.063
Max fish width	BM1	-3200.3	275.9	1602.1	0.001	-	-	-	-	-
	BMS	-3221	255.2	1613.5	0.001	0.001	-	-	-	-
	OU1	-3452.2	24	1730.1	0.001	-	0.033	-	-0.006	-
	OUM	-3459.1	17.1	1734.6	0.001	-	0.032	-	0.089	0.173
	**OUMV**	**-3476.2**	**0**	1744.1	0.001	0.001	0.035	0.027	0.089	0.173
Min caudalpeduncle depth	BM1	-154.8	112.7	79.4	0.002	-	-	-	-	-
	BMS	-235.4	32.1	120.7	0.003	0.002	-	-	-	-
	OU1	-171.8	95.7	89.9	0.003	-	0.441	-	855046.25	-
	OUM	-175.3	92.2	92.7	0.003	-	0.438	-	0.842	2.153
	**OUMV**	**-267.5**	**0**	139.8	0.003	0.002	0.433	0.223	0.658	1.588
Min caudalpeduncle width	BM1	2374.5	429.2	-1185.2	0.007	-	-	-	-	-
	BMS	2337.4	392.1	-1165.7	0.008	0.006	-	-	-	-
	OU1	2022	76.7	-1007	0.01	-	0.317	-	0.087	-
	OUM	2021.1	75.8	-1005.5	0.01	-	0.315	-	0.168	0.451
	**OUMV**	**1945.3**	**0**	-966.6	0.012	0.007	0.348	0.191	0.199	0.512
Mouth width	BM1	2037.3	695.4	-1016.6	0.006	-	-	-	-	-
	BMS	2033.9	691.9	-1013.9	0.007	0.006	-	-	-	-
	OU1	1365.8	23.9	-678.9	0.01	-	0.168	-	0.09	-
	OUM	1351.2	9.3	-670.6	0.01	-	0.166	-	0.173	0.094
	**OUMV**	**1341.9**	**0**	-664.9	0.011	0.009	0.176	0.141	0.175	0.097
Standard length	BM1	-3175	22.7	1589.5	0.001	-	-	-	-	-
	BMS	-3191.7	6	1598.8	0.001	0.001	-	-	-	-
	OU1	-3173.5	24.2	1590.8	0.001	-	0.36	-	4037296.335	-
	OUM	-3181.2	16.5	1595.6	0.001	-	0.326	-	-1.112	-4.84
	**OUMV**	**-3197.7**	**0**	1604.9	0.001	0.001	0.307	0.231	-0.978	-4.098
