# Supplementary material for: The Effect of Locomotion Mode on Body Shape Evolution in Teleost Fishes
Source: Integr Org Biol. 2021 May 18;3(1):obab016. doi: 10.1093/iob/obab016 (PMC8341890; doi:10.1093/iob/obab016)
Supplement: obab016_Supplementary_Data [file obab016_supplementary_data.zip › TableS1.pdf]

Species	Family	Locomotion Mode
Acanthurus achilles	Acanthuridae	MPF
Acanthurus bahianus	Acanthuridae	MPF
Acanthurus blochii	Acanthuridae	MPF
Acanthurus chirurgus	Acanthuridae	MPF
Acanthurus coeruleus	Acanthuridae	MPF
Acanthurus dussumieri	Acanthuridae	MPF
Acanthurus guttatus	Acanthuridae	MPF
Acanthurus japonicus	Acanthuridae	MPF
Acanthurus lineatus	Acanthuridae	MPF
Acanthurus mata	Acanthuridae	MPF
Acanthurus monroviae	Acanthuridae	MPF
Acanthurus nigricans	Acanthuridae	MPF
Acanthurus nigricauda	Acanthuridae	MPF
Acanthurus nigrofuscus	Acanthuridae	MPF
Acanthurus nigroris	Acanthuridae	MPF
Acanthurus nubilus	Acanthuridae	MPF
Acanthurus olivaceus	Acanthuridae	MPF
Acanthurus pyroferus	Acanthuridae	MPF
Acanthurus thompsoni	Acanthuridae	MPF
Acanthurus triostegus	Acanthuridae	MPF
Acanthurus xanthopterus	Acanthuridae	MPF
Ctenochaetus binotatus	Acanthuridae	MPF
Ctenochaetus hawaiiensis	Acanthuridae	MPF
Ctenochaetus striatus	Acanthuridae	MPF
Ctenochaetus strigosus	Acanthuridae	MPF
Naso annulatus	Acanthuridae	BCF
Naso brachycentron	Acanthuridae	BCF
Naso brevirostris	Acanthuridae	BCF
Naso caeruleacauda	Acanthuridae	BCF
Naso hexacanthus	Acanthuridae	BCF
Naso lituratus	Acanthuridae	BCF
Naso lopezi	Acanthuridae	BCF
Naso minor	Acanthuridae	BCF
Naso thynnoides	Acanthuridae	BCF
Naso tuberosus	Acanthuridae	BCF
Naso unicornis	Acanthuridae	BCF
Naso vlamingii	Acanthuridae	BCF
Paracanthurus hepatus	Acanthuridae	MPF
Prionurus laticlavius	Acanthuridae	MPF
Prionurus maculatus	Acanthuridae	MPF
Prionurus microlepidotus	Acanthuridae	MPF
Prionurus punctatus	Acanthuridae	MPF
Prionurus scalprum	Acanthuridae	MPF
Zebrasoma flavescens	Acanthuridae	MPF
Zebrasoma scopas	Acanthuridae	MPF
Zebrasoma velifer	Acanthuridae	MPF
Synagrops bellus	Acropomatidae	BCF
Synagrops japonicus	Acropomatidae	BCF
Synagrops philippinensis	Acropomatidae	BCF
Synagrops spinosus	Acropomatidae	BCF
Albula vulpes	Albulidae	BCF

(continued)

Species	Family	Locomotion Mode
Alepisaurus brevirostris	Alepisauridae	BCF
Alepisaurus ferox	Alepisauridae	BCF
Alepocephalus agassizii	Alepocephalidae	BCF
Alepocephalus bairdii	Alepocephalidae	BCF
Alepocephalus bicolor	Alepocephalidae	BCF
Alepocephalus owstoni	Alepocephalidae	BCF
Alepocephalus tenebrosus	Alepocephalidae	BCF
Bajacalifornia burragei	Alepocephalidae	BCF
Rouleina attrita	Alepocephalidae	BCF
Talismania bifurcata	Alepocephalidae	BCF
Xenodermichthys copei	Alepocephalidae	BCF
Anoplogaster cornuta	Anoplogastridae	BCF
Anoplopoma fimbria	Anoplopomatidae	BCF
Anotopterus pharao	Anotopteridae	BCF
Apogon aurolineatus	Apogonidae	BCF
Apogon binotatus	Apogonidae	BCF
Apogon campbelli	Apogonidae	BCF
Apogon caudicinctus	Apogonidae	BCF
Apogon coccineus	Apogonidae	BCF
Apogon doryssa	Apogonidae	BCF
Apogon dovii	Apogonidae	BCF
Apogon erythrinus	Apogonidae	BCF
Apogon guadalupensis	Apogonidae	BCF
Apogon imberbis	Apogonidae	BCF
Apogon indicus	Apogonidae	BCF
Apogon lachneri	Apogonidae	BCF
Apogon maculatus	Apogonidae	BCF
Apogon pacificus	Apogonidae	BCF
Apogon phenax	Apogonidae	BCF
Apogon pillionatus	Apogonidae	BCF
Apogon planifrons	Apogonidae	BCF
Apogon pseudomaculatus	Apogonidae	BCF
Apogon robinsi	Apogonidae	BCF
Apogon semiornatus	Apogonidae	BCF
Apogon townsendi	Apogonidae	BCF
Apogonichthyoides nigripinnis	Apogonidae	BCF
Apogonichthyoides taeniatus	Apogonidae	BCF
Astrapogon alutus	Apogonidae	BCF
Astrapogon puncticulatus	Apogonidae	BCF
Astrapogon stellatus	Apogonidae	BCF
Cercamia eremia	Apogonidae	BCF
Cheilodipterus artus	Apogonidae	BCF
Cheilodipterus isostigmus	Apogonidae	BCF
Cheilodipterus macrodon	Apogonidae	BCF
Cheilodipterus quinquelineatus	Apogonidae	BCF
Fowleria aurita	Apogonidae	BCF
Fowleria isostigma	Apogonidae	BCF
Fowleria marmorata	Apogonidae	BCF
Fowleria vaiulae	Apogonidae	BCF
Fowleria variegata	Apogonidae	BCF
Gymnapogon urospilotus	Apogonidae	BCF

(continued)

Species	Family	Locomotion Mode
Nectamia bandanensis	Apogonidae	BCF
Nectamia fusca	Apogonidae	BCF
Nectamia savayensis	Apogonidae	BCF
Ostorhinchus angustatus	Apogonidae	BCF
Ostorhinchus apogonoides	Apogonidae	BCF
Ostorhinchus aureus	Apogonidae	BCF
Ostorhinchus cookii	Apogonidae	BCF
Ostorhinchus endekataenia	Apogonidae	BCF
Ostorhinchus fasciatus	Apogonidae	BCF
Ostorhinchus holotaenia	Apogonidae	BCF
Ostorhinchus nigrofasciatus	Apogonidae	BCF
Ostorhinchus novemfasciatus	Apogonidae	BCF
Ostorhinchus sealei	Apogonidae	BCF
Ostorhinchus taeniophorus	Apogonidae	BCF
Phaeoptyx conklini	Apogonidae	BCF
Phaeoptyx pigmentaria	Apogonidae	BCF
Phaeoptyx xenus	Apogonidae	BCF
Pristiapogon exostigma	Apogonidae	BCF
Pristiapogon fraenatus	Apogonidae	BCF
Pristiapogon kallopterus	Apogonidae	BCF
Pristiapogon taeniopterus	Apogonidae	BCF
Pristicon trimaculatus	Apogonidae	BCF
Pseudamia gelatinosa	Apogonidae	BCF
Rhabdamia gracilis	Apogonidae	BCF
Sphaeramia nematoptera	Apogonidae	BCF
Sphaeramia orbicularis	Apogonidae	BCF
Zoramia fragilis	Apogonidae	BCF
Anoplocapros inermis	Aracanidae	MPF
Aracana ornata	Aracanidae	MPF
Kentrocapros rosapinto	Aracanidae	MPF
Argentina silus	Argentinidae	BCF
Argentina striata	Argentinidae	BCF
Amissidens hainesi	Ariidae	BCF
Ariopsis felis	Ariidae	BCF
Arius arius	Ariidae	BCF
Arius subrostratus	Ariidae	BCF
Arius venosus	Ariidae	BCF
Bagre bagre	Ariidae	BCF
Bagre marinus	Ariidae	BCF
Bagre panamensis	Ariidae	BCF
Cathorops multiradiatus	Ariidae	BCF
Cathorops spixii	Ariidae	BCF
Galeichthys ater	Ariidae	BCF
Galeichthys feliceps	Ariidae	BCF
Galeichthys peruvianus	Ariidae	BCF
Genidens genidens	Ariidae	BCF
Hexanematichthys mastersi	Ariidae	BCF
Nemapteryx caelata	Ariidae	BCF
Notarius grandicassis	Ariidae	BCF
Plicofollis argyropleuron	Ariidae	BCF
Plicofollis tenuispinis	Ariidae	BCF

(continued)

Species	Family	Locomotion Mode
Plicofollis tonggol	Ariidae	BCF
Ariomma bondi	Ariommatidae	BCF
Ariomma indicum	Ariommatidae	BCF
Ariomma melanum	Ariommatidae	BCF
Arripis georgianus	Arripidae	BCF
Arripis trutta	Arripidae	BCF
Ateleopus japonicus	Ateleopodidae	BCF
Ijimaia antillarum	Ateleopodidae	BCF
Ijimaia loppei	Ateleopodidae	BCF
Atherinomorus endrachtensis	Atherinidae	BCF
Atherinomorus vaigiensis	Atherinidae	BCF
Craterocephalus honoriae	Atherinidae	BCF
Hypoatherina harringtonensis	Atherinidae	BCF
Hypoatherina temminckii	Atherinidae	BCF
Atherinops affinis	Atherinopsidae	BCF
Leuresthes tenuis	Atherinopsidae	BCF
Membras martinica	Atherinopsidae	BCF
Menidia menidia	Atherinopsidae	BCF
Aulopus filamentosus	Aulopidae	BCF
Hime japonica	Aulopidae	BCF
Aulichthys japonicus	Aulorhynchidae	MPF
Aulorhynchus flavidus	Aulorhynchidae	MPF
Aulostomus chinensis	Aulostomidae	MPF
Aulostomus maculatus	Aulostomidae	MPF
Aulostomus strigosus	Aulostomidae	MPF
Abalistes stellatus	Balistidae	BCF
Balistapus undulatus	Balistidae	MPF
Balistes capriscus	Balistidae	MPF
Balistes polylepis	Balistidae	MPF
Balistes punctatus	Balistidae	MPF
Balistes vetula	Balistidae	MPF
Balistoides conspicillum	Balistidae	MPF
Balistoides viridescens	Balistidae	MPF
Canthidermis maculata	Balistidae	MPF
Canthidermis sufflamen	Balistidae	MPF
Melichthys indicus	Balistidae	MPF
Melichthys niger	Balistidae	MPF
Melichthys vidua	Balistidae	MPF
Odonus niger	Balistidae	MPF
Pseudobalistes flavimarginatus	Balistidae	MPF
Pseudobalistes fuscus	Balistidae	MPF
Pseudobalistes naufragium	Balistidae	MPF
Rhinecanthus aculeatus	Balistidae	MPF
Rhinecanthus assasi	Balistidae	MPF
Rhinecanthus lunula	Balistidae	MPF
Rhinecanthus rectangulus	Balistidae	MPF
Rhinecanthus verrucosus	Balistidae	MPF
Sufflamen albicaudatum	Balistidae	MPF
Sufflamen bursa	Balistidae	MPF
Sufflamen chrysopterum	Balistidae	MPF
Sufflamen fraenatum	Balistidae	MPF

(continued)

Species	Family	Locomotion Mode
Sufflamen verres	Balistidae	MPF
Xanthichthys auromarginatus	Balistidae	MPF
Xanthichthys lineopunctatus	Balistidae	MPF
Xanthichthys mento	Balistidae	MPF
Xanthichthys ringens	Balistidae	MPF
Banjos banjos	Banjosidae	BCF
Barbourisia rufa	Barbourisiidae	BCF
Bathyclupea argentea	Bathyclupeidae	BCF
Bathylagus antarcticus	Bathylagidae	BCF
Bathylagus euryops	Bathylagidae	BCF
Bathylagus tenuis	Bathylagidae	BCF
Dolicholagus longirostris	Bathylagidae	BCF
Lipolagus ochotensis	Bathylagidae	BCF
Pseudobathylagus milleri	Bathylagidae	BCF
Bathysaurus ferox	Bathysauridae	BCF
Ablennes hians	Belonidae	BCF
Belone belone	Belonidae	BCF
Belone svetovidovi	Belonidae	BCF
Petalichthys capensis	Belonidae	BCF
Platybelone argalus argalus	Belonidae	BCF
Strongylura anastomella	Belonidae	BCF
Strongylura incisa	Belonidae	BCF
Strongylura leiura	Belonidae	BCF
Strongylura scapularis	Belonidae	BCF
Strongylura senegalensis	Belonidae	BCF
Strongylura strongylura	Belonidae	BCF
Tylosurus acus acus	Belonidae	BCF
Tylosurus acus imperialis	Belonidae	BCF
Tylosurus acus rafale	Belonidae	BCF
Tylosurus crocodilus crocodilus	Belonidae	BCF
Tylosurus gavialoides	Belonidae	BCF
Tylosurus pacificus	Belonidae	BCF
Tylosurus punctulatus	Belonidae	BCF
Bembrops heterurus	Bembropidae	BCF
Beryx splendens	Berycidae	MPF
Centroberyx affinis	Berycidae	MPF
Centroberyx gerrardi	Berycidae	MPF
Meiacanthus grammistes	Blenniidae	BCF
Meiacanthus smithi	Blenniidae	BCF
Brama brama	Bramidae	BCF
Pterycombus brama	Bramidae	BCF
Pterycombus petersii	Bramidae	BCF
Taractichthys steindachneri	Bramidae	BCF
Bregmaceros cantori	Bregmacerotidae	BCF
Brosmophycis marginata	Bythitidae	BCF
Diancistrus fuscus	Bythitidae	BCF
Caesio caerulaurea	Caesionidae	BCF
Caesio lunaris	Caesionidae	BCF
Caesio xanthonota	Caesionidae	BCF
Dipterygonotus balteatus	Caesionidae	BCF
Gymnocaesio gymnoptera	Caesionidae	BCF

(continued)

Species	Family	Locomotion Mode
Pterocaesio digramma	Caesionidae	BCF
Pterocaesio marri	Caesionidae	BCF
Pterocaesio pisang	Caesionidae	BCF
Antigonia capros	Caproidae	MPF
Antigonia combatia	Caproidae	MPF
Antigonia rubescens	Caproidae	MPF
Capros aper	Caproidae	MPF
Alectis indica	Carangidae	BCF
Carangoides bajad	Carangidae	BCF
Carangoides chrysophrys	Carangidae	BCF
Carangoides ferdau	Carangidae	BCF
Carangoides malabaricus	Carangidae	BCF
Carangoides otrynter	Carangidae	BCF
Caranx bucculentus	Carangidae	BCF
Caranx caninus	Carangidae	BCF
Caranx crysos	Carangidae	BCF
Caranx hippos	Carangidae	BCF
Caranx ignobilis	Carangidae	BCF
Caranx lugubris	Carangidae	BCF
Caranx melampygus	Carangidae	BCF
Caranx papuensis	Carangidae	BCF
Caranx ruber	Carangidae	BCF
Chloroscombrus chrysurus	Carangidae	BCF
Chloroscombrus orqueta	Carangidae	BCF
Decapterus kurroides	Carangidae	BCF
Decapterus macarellus	Carangidae	BCF
Decapterus muroadsi	Carangidae	BCF
Decapterus punctatus	Carangidae	BCF
Decapterus russelli	Carangidae	BCF
Elagatis bipinnulata	Carangidae	BCF
Gnathanodon speciosus	Carangidae	BCF
Hemicaranx leucurus	Carangidae	BCF
Hemicaranx zelotes	Carangidae	BCF
Megalaspis cordyla	Carangidae	BCF
Oligoplites altus	Carangidae	BCF
Oligoplites saurus	Carangidae	BCF
Parastromateus niger	Carangidae	BCF
Pseudocaranx chilensis	Carangidae	BCF
Pseudocaranx dentex	Carangidae	BCF
Scomberoides commersonnianus	Carangidae	BCF
Scomberoides lysan	Carangidae	BCF
Selar boops	Carangidae	BCF
Selar crumenophthalmus	Carangidae	BCF
Selaroides leptolepis	Carangidae	BCF
Selene brevoortii	Carangidae	BCF
Selene orstedii	Carangidae	BCF
Selene vomer	Carangidae	BCF
Seriola dumerili	Carangidae	BCF
Seriola fasciata	Carangidae	BCF
Seriola lalandi	Carangidae	BCF
Seriola quinqueradiata	Carangidae	BCF

(continued)

Species	Family	Locomotion Mode
Seriola rivoliana	Carangidae	BCF
Seriola zonata	Carangidae	BCF
Trachinotus baillonii	Carangidae	BCF
Trachinotus carolinus	Carangidae	BCF
Trachinotus falcatus	Carangidae	BCF
Trachinotus goodei	Carangidae	BCF
Trachinotus ovatus	Carangidae	BCF
Trachinotus rhodopus	Carangidae	BCF
Trachurus capensis	Carangidae	BCF
Trachurus declivis	Carangidae	BCF
Trachurus japonicus	Carangidae	BCF
Trachurus lathami	Carangidae	BCF
Trachurus mediterraneus	Carangidae	BCF
Trachurus murphyi	Carangidae	BCF
Trachurus novaezelandiae	Carangidae	BCF
Trachurus picturatus	Carangidae	BCF
Trachurus trachurus	Carangidae	BCF
Uraspis helvola	Carangidae	BCF
Spicara axillaris	Centracanthidae	BCF
Spicara maena	Centracanthidae	BCF
Spicara smaris	Centracanthidae	BCF
Aeoliscus strigatus	Centriscidae	MPF
Macroramphosus gracilis	Centriscidae	MPF
Macroramphosus scolopax	Centriscidae	MPF
Notopogon fernandezianus	Centriscidae	MPF
Centrolophus niger	Centrolophidae	BCF
Hyperoglyphe perciformis	Centrolophidae	BCF
Icichthys lockingtoni	Centrolophidae	BCF
Psenopsis anomala	Centrolophidae	BCF
Psenopsis cyanea	Centrolophidae	BCF
Schedophilus medusophagus	Centrolophidae	BCF
Schedophilus ovalis	Centrolophidae	BCF
Seriolella brama	Centrolophidae	BCF
Seriolella punctata	Centrolophidae	BCF
Centropomus armatus	Centropomidae	BCF
Centropomus robalito	Centropomidae	BCF
Centropomus unionensis	Centropomidae	BCF
Ceratias holboelli	Ceratiidae	BCF
Cryptopsaras couesii	Ceratiidae	BCF
Cetostoma regani	Cetomimidae	MPF
Ditropichthys storeri	Cetomimidae	MPF
Amphichaetodon howensis	Chaetodontidae	MPF
Amphichaetodon melbae	Chaetodontidae	MPF
Chaetodon adiergastos	Chaetodontidae	MPF
Chaetodon aureofasciatus	Chaetodontidae	MPF
Chaetodon auriga	Chaetodontidae	MPF
Chaetodon auripes	Chaetodontidae	MPF
Chaetodon austriacus	Chaetodontidae	MPF
Chaetodon baronessa	Chaetodontidae	MPF
Chaetodon bennetti	Chaetodontidae	MPF
Chaetodon blackburnii	Chaetodontidae	MPF

(continued)

Species	Family	Locomotion Mode
Chaetodon capistratus	Chaetodontidae	MPF
Chaetodon citrinellus	Chaetodontidae	MPF
Chaetodon collare	Chaetodontidae	MPF
Chaetodon decussatus	Chaetodontidae	MPF
Chaetodon dolosus	Chaetodontidae	MPF
Chaetodon ephippium	Chaetodontidae	MPF
Chaetodon falcula	Chaetodontidae	MPF
Chaetodon fasciatus	Chaetodontidae	MPF
Chaetodon flavirostris	Chaetodontidae	MPF
Chaetodon fremblii	Chaetodontidae	MPF
Chaetodon guentheri	Chaetodontidae	MPF
Chaetodon guttatissimus	Chaetodontidae	MPF
Chaetodon hoefleri	Chaetodontidae	MPF
Chaetodon kleinii	Chaetodontidae	MPF
Chaetodon larvatus	Chaetodontidae	MPF
Chaetodon lineolatus	Chaetodontidae	MPF
Chaetodon lunula	Chaetodontidae	MPF
Chaetodon madagaskariensis	Chaetodontidae	MPF
Chaetodon melannotus	Chaetodontidae	MPF
Chaetodon mertensii	Chaetodontidae	MPF
Chaetodon meyeri	Chaetodontidae	MPF
Chaetodon miliaris	Chaetodontidae	MPF
Chaetodon multicinctus	Chaetodontidae	MPF
Chaetodon ocellatus	Chaetodontidae	MPF
Chaetodon ocellicaudus	Chaetodontidae	MPF
Chaetodon octofasciatus	Chaetodontidae	MPF
Chaetodon ornatissimus	Chaetodontidae	MPF
Chaetodon oxycephalus	Chaetodontidae	MPF
Chaetodon pelewensis	Chaetodontidae	MPF
Chaetodon plebeius	Chaetodontidae	MPF
Chaetodon punctatofasciatus	Chaetodontidae	MPF
Chaetodon quadrimaculatus	Chaetodontidae	MPF
Chaetodon rafflesii	Chaetodontidae	MPF
Chaetodon rainfordi	Chaetodontidae	MPF
Chaetodon reticulatus	Chaetodontidae	MPF
Chaetodon robustus	Chaetodontidae	MPF
Chaetodon sanctaehelenae	Chaetodontidae	MPF
Chaetodon sedentarius	Chaetodontidae	MPF
Chaetodon selene	Chaetodontidae	MPF
Chaetodon semeion	Chaetodontidae	MPF
Chaetodon semilarvatus	Chaetodontidae	MPF
Chaetodon speculum	Chaetodontidae	MPF
Chaetodon striatus	Chaetodontidae	MPF
Chaetodon trichrous	Chaetodontidae	MPF
Chaetodon trifascialis	Chaetodontidae	MPF
Chaetodon trifasciatus	Chaetodontidae	MPF
Chaetodon ulietensis	Chaetodontidae	MPF
Chaetodon unimaculatus	Chaetodontidae	MPF
Chaetodon vagabundus	Chaetodontidae	MPF
Chaetodon xanthurus	Chaetodontidae	MPF
Chaetodon zanzibarensis	Chaetodontidae	MPF

(continued)

Species	Family	Locomotion Mode
Chelmon marginalis	Chaetodontidae	MPF
Chelmon rostratus	Chaetodontidae	MPF
Coradion chrysozonus	Chaetodontidae	MPF
Coradion melanopus	Chaetodontidae	MPF
Forcipiger flavissimus	Chaetodontidae	MPF
Forcipiger longirostris	Chaetodontidae	MPF
Hemitaurichthys polylepis	Chaetodontidae	MPF
Hemitaurichthys thompsoni	Chaetodontidae	MPF
Hemitaurichthys zoster	Chaetodontidae	MPF
Heniochus acuminatus	Chaetodontidae	MPF
Heniochus chrysostomus	Chaetodontidae	MPF
Heniochus diphreutes	Chaetodontidae	MPF
Heniochus monoceros	Chaetodontidae	MPF
Heniochus singularius	Chaetodontidae	MPF
Heniochus varius	Chaetodontidae	MPF
Johnrandallia nigrirostris	Chaetodontidae	MPF
Prognathodes aculeatus	Chaetodontidae	MPF
Prognathodes aya	Chaetodontidae	MPF
Prognathodes marcellae	Chaetodontidae	MPF
Chaenocephalus aceratus	Channichthyidae	MPF
Champsocephalus esox	Channichthyidae	MPF
Channichthys rhinoceratus	Channichthyidae	MPF
Chionodraco hamatus	Channichthyidae	MPF
Chionodraco myersi	Channichthyidae	MPF
Chionodraco rastrospinosus	Channichthyidae	MPF
Cryodraco antarcticus	Channichthyidae	MPF
Dacodraco hunteri	Channichthyidae	MPF
Pseudochaenichthys georgianus	Channichthyidae	MPF
Cheilodactylus fuscus	Cheilodactylidae	BCF
Cheilodactylus variegatus	Cheilodactylidae	BCF
Cheilodactylus zonatus	Cheilodactylidae	BCF
Chirodactylus brachydactylus	Cheilodactylidae	BCF
Nemadactylus bergi	Cheilodactylidae	BCF
Nemadactylus macropterus	Cheilodactylidae	BCF
Chiasmodon niger	Chiasmodontidae	BCF
Dysalotus alcocki	Chiasmodontidae	BCF
Kali indica	Chiasmodontidae	BCF
Kali kerberti	Chiasmodontidae	BCF
Kali macrura	Chiasmodontidae	BCF
Chirocentrus dorab	Chirocentridae	BCF
Amblygaster clupeoides	Clupeidae	BCF
Brevoortia aurea	Clupeidae	BCF
Brevoortia patronus	Clupeidae	BCF
Brevoortia smithi	Clupeidae	BCF
Brevoortia tyrannus	Clupeidae	BCF
Clupea harengus	Clupeidae	BCF
Harengula clupeola	Clupeidae	BCF
Harengula humeralis	Clupeidae	BCF
Harengula jaguana	Clupeidae	BCF
Harengula thrissina	Clupeidae	BCF
Jenkinsia lamprotaenia	Clupeidae	BCF

(continued)

Species	Family	Locomotion Mode
Konosirus punctatus	Clupeidae	BCF
Nematalosa japonica	Clupeidae	BCF
Opisthonema libertate	Clupeidae	BCF
Opisthonema medirastre	Clupeidae	BCF
Opisthonema oglinum	Clupeidae	BCF
Sardinella albella	Clupeidae	BCF
Sardinella aurita	Clupeidae	BCF
Sardinella brasiliensis	Clupeidae	BCF
Sardinella fimbriata	Clupeidae	BCF
Sardinella gibbosa	Clupeidae	BCF
Sardinella hualiensis	Clupeidae	BCF
Sardinella jussieu	Clupeidae	BCF
Sardinella lemuru	Clupeidae	BCF
Sardinella longiceps	Clupeidae	BCF
Sardinella maderensis	Clupeidae	BCF
Sardinella melanura	Clupeidae	BCF
Sardinella zunasi	Clupeidae	BCF
Sardinops sagax	Clupeidae	BCF
Spratelloides delicatulus	Clupeidae	BCF
Spratelloides gracilis	Clupeidae	BCF
Spratelloides robustus	Clupeidae	BCF
Sprattus sprattus	Clupeidae	BCF
Coloconger cadenati	Colocongridae	BCF
Bassanago albescens	Congridae	BCF
Bathycongrus retrotinctus	Congridae	BCF
Bathycongrus wallacei	Congridae	BCF
Conger conger	Congridae	BCF
Conger myriaster	Congridae	BCF
Conger oceanicus	Congridae	BCF
Gnathophis bathytopos	Congridae	BCF
Paraconger notialis	Congridae	BCF
Uroconger lepturus	Congridae	BCF
Uroconger syringinus	Congridae	BCF
Xenomystax congroides	Congridae	BCF
Coryphaena hippurus	Coryphaenidae	BCF
Cryptacanthodes maculatus	Cryptacanthodidae	BCF
Floridichthys carpio	Cyprinodontidae	BCF
Cyttus australis	Cyttidae	MPF
Derichthys serpentinus	Derichthyidae	BCF
Nessorhamphus ingolfianus	Derichthyidae	BCF
Dinoperca petersi	Dinopercidae	BCF
Allomycterus pilatus	Diodontidae	MPF
Chilomycterus antennatus	Diodontidae	MPF
Chilomycterus antillarum	Diodontidae	MPF
Chilomycterus reticulatus	Diodontidae	MPF
Chilomycterus schoepfii	Diodontidae	MPF
Chilomycterus spinosus spinosus	Diodontidae	MPF
Diodon holocanthus	Diodontidae	MPF
Diodon hystrix	Diodontidae	MPF
Diodon liturosus	Diodontidae	MPF
Diodon nicthemerus	Diodontidae	MPF

(continued)

Species	Family	Locomotion Mode
Diretmichthys parini	Diretmidae	BCF
Diretmus argenteus	Diretmidae	BCF
Drepane africana	Drepaneidae	BCF
Drepane longimana	Drepaneidae	BCF
Dussumieria elopsoides	Dussumieriidae	BCF
Etrumeus acuminatus	Dussumieriidae	BCF
Etrumeus makiawa	Dussumieriidae	BCF
Etrumeus micropus	Dussumieriidae	BCF
Etrumeus sadina	Dussumieriidae	BCF
Eleginops maclovinus	Eleginopsidae	BCF
Erotelis armiger	Eleotridae	MPF
Elops affinis	Elopidae	BCF
Elops machnata	Elopidae	BCF
Elops saurus	Elopidae	BCF
Amphistichus rhodoterus	Embiotocidae	MPF
Brachyistius frenatus	Embiotocidae	MPF
Cymatogaster aggregata	Embiotocidae	MPF
Ditrema temminckii temminckii	Embiotocidae	MPF
Embiotoca jacksoni	Embiotocidae	MPF
Embiotoca lateralis	Embiotocidae	MPF
Hyperprosopon anale	Embiotocidae	MPF
Hyperprosopon argenteum	Embiotocidae	MPF
Hypsurus caryi	Embiotocidae	MPF
Micrometrus minimus	Embiotocidae	MPF
Neoditrema ransonnetii	Embiotocidae	MPF
Phanerodon furcatus	Embiotocidae	MPF
Rhacochilus vacca	Embiotocidae	MPF
Zalembius rosaceus	Embiotocidae	MPF
Emmelichthys nitidus nitidus	Emmelichthyidae	BCF
Erythrocles monodi	Emmelichthyidae	BCF
Erythrocles schlegelii	Emmelichthyidae	BCF
Plagiogeneion rubiginosum	Emmelichthyidae	BCF
Anchoa cayorum	Engraulidae	BCF
Anchoa choerostoma	Engraulidae	BCF
Anchoa colonensis	Engraulidae	BCF
Anchoa compressa	Engraulidae	BCF
Anchoa cubana	Engraulidae	BCF
Anchoa delicatissima	Engraulidae	BCF
Anchoa filifera	Engraulidae	BCF
Anchoa hepsetus	Engraulidae	BCF
Anchoa lamprotaenia	Engraulidae	BCF
Anchoa lyolepis	Engraulidae	BCF
Anchoa mundeoloides	Engraulidae	BCF
Anchoa nasus	Engraulidae	BCF
Anchoa panamensis	Engraulidae	BCF
Anchovia macrolepidota	Engraulidae	BCF
Anchoviella balboae	Engraulidae	BCF
Anchoviella brevirostris	Engraulidae	BCF
Cetengraulis edentulus	Engraulidae	BCF
Cetengraulis mysticetus	Engraulidae	BCF
Coilia ramcarati	Engraulidae	BCF

(continued)

Species	Family	Locomotion Mode
Encrasicholina devisi	Engraulidae	BCF
Engraulis anchoita	Engraulidae	BCF
Engraulis australis	Engraulidae	BCF
Engraulis encrasicolus	Engraulidae	BCF
Engraulis eurystole	Engraulidae	BCF
Engraulis japonicus	Engraulidae	BCF
Engraulis mordax	Engraulidae	BCF
Engraulis ringens	Engraulidae	BCF
Lycengraulis poeyi	Engraulidae	BCF
Setipinna taty	Engraulidae	BCF
Thryssa mystax	Engraulidae	BCF
Thryssa vitrirostris	Engraulidae	BCF
Enoplosus armatus	Enoplosidae	MPF
Chaetodipterus faber	Ephippidae	BCF
Ephippus orbis	Ephippidae	BCF
Platax batavianus	Ephippidae	BCF
Platax boersii	Ephippidae	BCF
Platax orbicularis	Ephippidae	BCF
Platax pinnatus	Ephippidae	BCF
Platax teira	Ephippidae	BCF
Tripterodon orbis	Ephippidae	BCF
Eurypharynx pelecanoides	Eurypharyngidae	BCF
Coccorella atlantica	Evermannellidae	BCF
Evermannella balbo	Evermannellidae	BCF
Evermannella indica	Evermannellidae	BCF
Cheilopogon abei	Exocoetidae	BCF
Cheilopogon antoncichi	Exocoetidae	BCF
Cheilopogon atrisignis	Exocoetidae	BCF
Cheilopogon cyanopterus	Exocoetidae	BCF
Cheilopogon dorsomacula	Exocoetidae	BCF
Cheilopogon exsiliens	Exocoetidae	BCF
Cheilopogon furcatus	Exocoetidae	BCF
Cheilopogon spilonotopterus	Exocoetidae	BCF
Cheilopogon unicolor	Exocoetidae	BCF
Cheilopogon xenopterus	Exocoetidae	BCF
Cypselurus angusticeps	Exocoetidae	BCF
Cypselurus callopterus	Exocoetidae	BCF
Cypselurus hexazona	Exocoetidae	BCF
Cypselurus poecilopterus	Exocoetidae	BCF
Exocoetus obtusirostris	Exocoetidae	BCF
Exocoetus volitans	Exocoetidae	BCF
Fodiator acutus	Exocoetidae	BCF
Fodiator rostratus	Exocoetidae	BCF
Hirundichthys affinis	Exocoetidae	BCF
Hirundichthys albimaculatus	Exocoetidae	BCF
Hirundichthys rondeletii	Exocoetidae	BCF
Hirundichthys speculiger	Exocoetidae	BCF
Parexocoetus brachypterus	Exocoetidae	BCF
Parexocoetus hillianus	Exocoetidae	BCF
Parexocoetus mento	Exocoetidae	BCF
Prognichthys gibbifrons	Exocoetidae	BCF

(continued)

Species	Family	Locomotion Mode
Prognichthys glaphyrae	Exocoetidae	BCF
Prognichthys occidentalis	Exocoetidae	BCF
Fistularia commersonii	Fistulariidae	MPF
Fistularia corneta	Fistulariidae	MPF
Fistularia petimba	Fistulariidae	MPF
Fundulus luciae	Fundulidae	BCF
Fundulus majalis	Fundulidae	BCF
Fundulus similis	Fundulidae	BCF
Boreogadus saida	Gadidae	BCF
Gadiculus argenteus	Gadidae	BCF
Gadus macrocephalus	Gadidae	BCF
Gadus morhua	Gadidae	BCF
Gadus ogac	Gadidae	BCF
Melanogrammus aeglefinus	Gadidae	BCF
Merlangius merlangus	Gadidae	BCF
Microgadus proximus	Gadidae	BCF
Micromesistius australis	Gadidae	BCF
Micromesistius poutassou	Gadidae	BCF
Pollachius pollachius	Gadidae	BCF
Pollachius virens	Gadidae	BCF
Theragra chalcogramma	Gadidae	BCF
Trisopterus esmarkii	Gadidae	BCF
Trisopterus luscus	Gadidae	BCF
Trisopterus minutus	Gadidae	BCF
Gasterosteus wheatlandi	Gasterosteidae	BCF
Spinachia spinachia	Gasterosteidae	BCF
Diplospinus multistriatus	Gempylidae	BCF
Gempylus serpens	Gempylidae	BCF
Lepidocybium flavobrunneum	Gempylidae	BCF
Nealotus tripes	Gempylidae	BCF
Neoepinnula americana	Gempylidae	BCF
Neoepinnula orientalis	Gempylidae	BCF
Nesiarchus nasutus	Gempylidae	BCF
Paradiplospinus antarcticus	Gempylidae	BCF
Promethichthys prometheus	Gempylidae	BCF
Rexea prometheoides	Gempylidae	BCF
Ruvettus pretiosus	Gempylidae	BCF
Thyrsites atun	Gempylidae	BCF
Diapterus auratus	Gerreidae	BCF
Diapterus peruvianus	Gerreidae	BCF
Gerres erythrourus	Gerreidae	BCF
Gerres macracanthus	Gerreidae	BCF
Gerres oyena	Gerreidae	BCF
Amblygobius albimaculatus	Gobiidae	MPF
Amblygobius nocturnus	Gobiidae	MPF
Amblygobius phalaena	Gobiidae	MPF
Amblygobius sphynx	Gobiidae	MPF
Aphia minuta	Gobiidae	BCF
Eviota spilota	Gobiidae	BCF
Gobiusculus flavescens	Gobiidae	BCF
Microgobius microlepis	Gobiidae	BCF

(continued)

Species	Family	Locomotion Mode
Pterogobius elapoides	Gobiidae	MPF
Pterogobius virgo	Gobiidae	BCF
Trimma benjamini	Gobiidae	MPF
Trimma flammeum	Gobiidae	MPF
Trimma haima	Gobiidae	MPF
Valenciennea longipinnis	Gobiidae	MPF
Valenciennea muralis	Gobiidae	MPF
Valenciennea parva	Gobiidae	MPF
Valenciennea puellaris	Gobiidae	MPF
Valenciennea sexguttata	Gobiidae	MPF
Valenciennea strigata	Gobiidae	MPF
Vanderhorstia ornatissima	Gobiidae	BCF
Gonorynchus greyi	Gonorynchidae	BCF
Bonapartia pedaliota	Gonostomatidae	BCF
Cyclothone acclinidens	Gonostomatidae	BCF
Cyclothone alba	Gonostomatidae	BCF
Cyclothone atraria	Gonostomatidae	BCF
Cyclothone microdon	Gonostomatidae	BCF
Cyclothone pallida	Gonostomatidae	BCF
Cyclothone pseudopallida	Gonostomatidae	BCF
Cyclothone signata	Gonostomatidae	BCF
Diplophos taenia	Gonostomatidae	BCF
Gonostoma atlanticum	Gonostomatidae	BCF
Gonostoma denudatum	Gonostomatidae	BCF
Gonostoma elongatum	Gonostomatidae	BCF
Margrethia obtusirostra	Gonostomatidae	BCF
Sigmops bathyphilus	Gonostomatidae	BCF
Sigmops ebelingi	Gonostomatidae	BCF
Sigmops gracilis	Gonostomatidae	BCF
Triplophos hemingi	Gonostomatidae	BCF
Gramma loreto	Grammatidae	MPF
Grammicolepis brachiusculus	Grammicolepididae	MPF
Xenolepidichthys dalgleishi	Grammicolepididae	MPF
Anisotremus caesius	Haemulidae	BCF
Anisotremus davidsonii	Haemulidae	BCF
Anisotremus interruptus	Haemulidae	BCF
Anisotremus moricandi	Haemulidae	BCF
Anisotremus scapularis	Haemulidae	BCF
Anisotremus surinamensis	Haemulidae	BCF
Anisotremus taeniatus	Haemulidae	BCF
Anisotremus virginicus	Haemulidae	BCF
Brachydeuterus auritus	Haemulidae	BCF
Diagramma pictum	Haemulidae	BCF
Genyatremus dovii	Haemulidae	BCF
Genyatremus luteus	Haemulidae	BCF
Genyatremus pacifici	Haemulidae	BCF
Haemulon album	Haemulidae	BCF
Haemulon aurolineatum	Haemulidae	BCF
Haemulon bonariense	Haemulidae	BCF
Haemulon boschmae	Haemulidae	BCF
Haemulon carbonarium	Haemulidae	BCF

(continued)

Species	Family	Locomotion Mode
Haemulon chrysargyreum	Haemulidae	BCF
Haemulon flaviguttatum	Haemulidae	BCF
Haemulon flavolineatum	Haemulidae	BCF
Haemulon macrostomum	Haemulidae	BCF
Haemulon maculicauda	Haemulidae	BCF
Haemulon melanurum	Haemulidae	BCF
Haemulon parra	Haemulidae	BCF
Haemulon plumierii	Haemulidae	BCF
Haemulon sciurus	Haemulidae	BCF
Haemulon scudderii	Haemulidae	BCF
Haemulon sexfasciatum	Haemulidae	BCF
Haemulon steindachneri	Haemulidae	BCF
Haemulon striatum	Haemulidae	BCF
Haemulopsis elongatus	Haemulidae	BCF
Haemulopsis nitidus	Haemulidae	BCF
Isacia conceptionis	Haemulidae	BCF
Microlepidotus inornatus	Haemulidae	BCF
Orthopristis chalceus	Haemulidae	BCF
Orthopristis chrysoptera	Haemulidae	BCF
Parapristipoma trilineatum	Haemulidae	BCF
Plectorhinchus albovittatus	Haemulidae	BCF
Plectorhinchus chaetodonoides	Haemulidae	BCF
Plectorhinchus chrysotaenia	Haemulidae	BCF
Plectorhinchus cinctus	Haemulidae	BCF
Plectorhinchus gaterinus	Haemulidae	BCF
Plectorhinchus lessonii	Haemulidae	BCF
Plectorhinchus lineatus	Haemulidae	BCF
Plectorhinchus macrolepis	Haemulidae	BCF
Plectorhinchus picus	Haemulidae	BCF
Pomadasys argyreus	Haemulidae	BCF
Pomadasys incisus	Haemulidae	BCF
Pomadasys kaakan	Haemulidae	BCF
Pomadasys maculatus	Haemulidae	BCF
Pomadasys olivaceus	Haemulidae	BCF
Pomadasys panamensis	Haemulidae	BCF
Pomadasys perotaei	Haemulidae	BCF
Xenichthys xanti	Haemulidae	BCF
Xenistius californiensis	Haemulidae	BCF
Aldrovandia affinis	Halosauridae	BCF
Aldrovandia phalacra	Halosauridae	BCF
Halosauropsis macrochir	Halosauridae	BCF
Euleptorhamphus viridis	Hemiramphidae	BCF
Hemiramphus balao	Hemiramphidae	BCF
Hemiramphus brasiliensis	Hemiramphidae	BCF
Hemiramphus far	Hemiramphidae	BCF
Hyporhamphus melanochir	Hemiramphidae	BCF
Hyporhamphus unifasciatus	Hemiramphidae	BCF
Oxyporhamphus micropterus micropterus	Hemiramphidae	BCF
Oxyporhamphus micropterus similis	Hemiramphidae	BCF
Blepsias cirrhosus	Hemitripteridae	BCF
Holocentrus adscensionis	Holocentridae	BCF

(continued)

Species	Family	Locomotion Mode
Holocentrus rufus	Holocentridae	BCF
Myripristis amaena	Holocentridae	BCF
Myripristis berndti	Holocentridae	BCF
Myripristis botche	Holocentridae	BCF
Myripristis chryseres	Holocentridae	BCF
Myripristis hexagona	Holocentridae	BCF
Myripristis jacobus	Holocentridae	BCF
Myripristis kuntee	Holocentridae	BCF
Myripristis leiognathus	Holocentridae	BCF
Myripristis murdjan	Holocentridae	BCF
Myripristis pralinia	Holocentridae	BCF
Myripristis randalli	Holocentridae	BCF
Myripristis violacea	Holocentridae	BCF
Myripristis vittata	Holocentridae	BCF
Neoniphon argenteus	Holocentridae	BCF
Neoniphon aurolineatus	Holocentridae	BCF
Neoniphon marianus	Holocentridae	BCF
Neoniphon opercularis	Holocentridae	BCF
Neoniphon sammara	Holocentridae	BCF
Ostichthys japonicus	Holocentridae	BCF
Ostichthys trachypoma	Holocentridae	BCF
Plectrypops lima	Holocentridae	BCF
Plectrypops retrospinis	Holocentridae	BCF
Sargocentron caudimaculatum	Holocentridae	BCF
Sargocentron cornutum	Holocentridae	BCF
Sargocentron coruscum	Holocentridae	BCF
Sargocentron diadema	Holocentridae	BCF
Sargocentron inaequalis	Holocentridae	BCF
Sargocentron ittodai	Holocentridae	BCF
Sargocentron melanospilos	Holocentridae	BCF
Sargocentron microstoma	Holocentridae	BCF
Sargocentron praslin	Holocentridae	BCF
Sargocentron punctatissimum	Holocentridae	BCF
Sargocentron rubrum	Holocentridae	BCF
Sargocentron seychellense	Holocentridae	BCF
Sargocentron spiniferum	Holocentridae	BCF
Sargocentron suborbitale	Holocentridae	BCF
Sargocentron tiere	Holocentridae	BCF
Sargocentron tiereoides	Holocentridae	BCF
Sargocentron vexillarium	Holocentridae	BCF
Sargocentron violaceum	Holocentridae	BCF
Sargocentron xantherythrum	Holocentridae	BCF
Icosteus aenigmaticus	Icosteidae	BCF
Haemulon vittatum	Inermiidae	BCF
Iso rhothophilus	Isonidae	BCF
Kajikia albida	Istiophoridae	BCF
Tetrapturus georgii	Istiophoridae	BCF
Tetrapturus pfluegeri	Istiophoridae	BCF
Kuhlia mugil	Kuhliidae	BCF
Kuhlia petiti	Kuhliidae	BCF
Atypichthys latus	Kyphosidae	BCF

(continued)

Species	Family	Locomotion Mode
Atypichthys strigatus	Kyphosidae	BCF
Girella cyanea	Kyphosidae	BCF
Girella elevata	Kyphosidae	BCF
Girella leonina	Kyphosidae	BCF
Girella nigricans	Kyphosidae	BCF
Girella punctata	Kyphosidae	BCF
Girella tricuspidata	Kyphosidae	BCF
Graus nigra	Kyphosidae	BCF
Kyphosus bigibbus	Kyphosidae	BCF
Kyphosus cinerascens	Kyphosidae	BCF
Kyphosus elegans	Kyphosidae	BCF
Kyphosus incisor	Kyphosidae	BCF
Kyphosus sectatrix	Kyphosidae	BCF
Kyphosus sydneyanus	Kyphosidae	BCF
Kyphosus vaigiensis	Kyphosidae	BCF
Medialuna californiensis	Kyphosidae	BCF
Scorpis lineolata	Kyphosidae	BCF
Scorpis violacea	Kyphosidae	BCF
Anampses caeruleopunctatus	Labridae	MPF
Anampses geographicus	Labridae	MPF
Anampses neoguinaicus	Labridae	MPF
Anampses twistii	Labridae	MPF
Austrolabrus maculatus	Labridae	MPF
Bodianus bilunulatus	Labridae	MPF
Bodianus mesothorax	Labridae	MPF
Bodianus perditio	Labridae	MPF
Bodianus rufus	Labridae	MPF
Bodianus scrofa	Labridae	MPF
Centrolabrus exoletus	Labridae	MPF
Centrolabrus trutta	Labridae	MPF
Cheilinus chlorourus	Labridae	MPF
Cheilinus fasciatus	Labridae	MPF
Cheilinus oxycephalus	Labridae	MPF
Cheilinus trilobatus	Labridae	MPF
Cheilinus undulatus	Labridae	MPF
Cheilio inermis	Labridae	MPF
Choerodon anchorago	Labridae	MPF
Choerodon azurio	Labridae	MPF
Choerodon fasciatus	Labridae	MPF
Choerodon oligacanthus	Labridae	MPF
Choerodon schoenleinii	Labridae	MPF
Cirrhilabrus lubbocki	Labridae	MPF
Clepticus parrae	Labridae	MPF
Coris aygula	Labridae	MPF
Coris batuensis	Labridae	MPF
Coris dorsomacula	Labridae	MPF
Coris flavovittata	Labridae	MPF
Coris formosa	Labridae	MPF
Coris gaimard	Labridae	MPF
Coris julis	Labridae	MPF
Coris picta	Labridae	MPF

(continued)

Species	Family	Locomotion Mode
Coris pictoides	Labridae	MPF
Ctenolabrus rupestris	Labridae	MPF
Cymolutes praetextatus	Labridae	MPF
Cymolutes torquatus	Labridae	MPF
Decodon melasma	Labridae	MPF
Diproctacanthus xanthurus	Labridae	MPF
Epibulus brevis	Labridae	MPF
Epibulus insidiator	Labridae	MPF
Gomphosus varius	Labridae	MPF
Halichoeres argus	Labridae	MPF
Halichoeres bathyphilus	Labridae	MPF
Halichoeres binotopsis	Labridae	MPF
Halichoeres biocellatus	Labridae	MPF
Halichoeres bivittatus	Labridae	MPF
Halichoeres chierchiae	Labridae	MPF
Halichoeres chloropterus	Labridae	MPF
Halichoeres chrysus	Labridae	MPF
Halichoeres cyanocephalus	Labridae	MPF
Halichoeres dispilus	Labridae	MPF
Halichoeres garnoti	Labridae	MPF
Halichoeres hartzfeldii	Labridae	MPF
Halichoeres hortulanus	Labridae	MPF
Halichoeres lapillus	Labridae	MPF
Halichoeres leucurus	Labridae	MPF
Halichoeres margaritaceus	Labridae	MPF
Halichoeres marginatus	Labridae	MPF
Halichoeres melanurus	Labridae	MPF
Halichoeres melasmapomus	Labridae	MPF
Halichoeres miniatus	Labridae	MPF
Halichoeres nebulosus	Labridae	MPF
Halichoeres nicholsi	Labridae	MPF
Halichoeres nigrescens	Labridae	MPF
Halichoeres notospilus	Labridae	MPF
Halichoeres ornatissimus	Labridae	MPF
Halichoeres papilionaceus	Labridae	MPF
Halichoeres pictus	Labridae	MPF
Halichoeres podostigma	Labridae	MPF
Halichoeres poeyi	Labridae	MPF
Halichoeres prosopeion	Labridae	MPF
Halichoeres radiatus	Labridae	MPF
Halichoeres richmondi	Labridae	MPF
Halichoeres scapularis	Labridae	MPF
Halichoeres semicinctus	Labridae	MPF
Halichoeres socialis	Labridae	MPF
Halichoeres solorensis	Labridae	MPF
Halichoeres trimaculatus	Labridae	MPF
Hemigymnus fasciatus	Labridae	MPF
Hemigymnus melapterus	Labridae	MPF
Hologymnosus annulatus	Labridae	MPF
Hologymnosus doliatus	Labridae	MPF
Iniistius aneitensis	Labridae	MPF

(continued)

Species	Family	Locomotion Mode
Labrichthys unilineatus	Labridae	MPF
Labroides bicolor	Labridae	MPF
Labroides dimidiatus	Labridae	MPF
Labroides rubrolabiatus	Labridae	MPF
Labropsis australis	Labridae	MPF
Labrus bergylta	Labridae	MPF
Labrus merula	Labridae	MPF
Labrus mixtus	Labridae	MPF
Lachnolaimus maximus	Labridae	MPF
Larabicus quadrilineatus	Labridae	MPF
Leptojulis cyanopleura	Labridae	MPF
Macropharyngodon bipartitus	Labridae	MPF
Macropharyngodon geoffroy	Labridae	MPF
Macropharyngodon meleagris	Labridae	MPF
Macropharyngodon negrosensis	Labridae	MPF
Macropharyngodon ornatus	Labridae	MPF
Malapterus reticulatus	Labridae	MPF
Notolabrus fucicola	Labridae	MPF
Notolabrus gymnogenis	Labridae	MPF
Notolabrus parilus	Labridae	MPF
Notolabrus tetricus	Labridae	MPF
Novaculichthys taeniourus	Labridae	MPF
Novaculoides macrolepidotus	Labridae	MPF
Ophthalmolepis lineolata	Labridae	MPF
Oxycheilinus bimaculatus	Labridae	MPF
Oxycheilinus celebicus	Labridae	MPF
Oxycheilinus digramma	Labridae	MPF
Oxycheilinus unifasciatus	Labridae	MPF
Oxyjulis californica	Labridae	MPF
Pictilabrus laticlavius	Labridae	MPF
Pseudocheilinus octotaenia	Labridae	MPF
Pseudocoris yamashiroi	Labridae	MPF
Pseudodax moluccanus	Labridae	MPF
Pseudojuloides atavai	Labridae	MPF
Pseudojuloides cerasinus	Labridae	MPF
Pseudolabrus gayi	Labridae	MPF
Pteragogus cryptus	Labridae	MPF
Pteragogus enneacanthus	Labridae	MPF
Semicossyphus darwini	Labridae	MPF
Semicossyphus pulcher	Labridae	MPF
Stethojulis albovittata	Labridae	MPF
Stethojulis balteata	Labridae	MPF
Stethojulis bandanensis	Labridae	MPF
Stethojulis trilineata	Labridae	MPF
Symphodus cinereus	Labridae	MPF
Symphodus melops	Labridae	MPF
Symphodus roissali	Labridae	MPF
Symphodus rostratus	Labridae	MPF
Symphodus tinca	Labridae	MPF
Tautoga onitis	Labridae	MPF
Tautogolabrus adspersus	Labridae	MPF

(continued)

Species	Family	Locomotion Mode
Thalassoma amblycephalum	Labridae	MPF
Thalassoma ascensionis	Labridae	MPF
Thalassoma ballieui	Labridae	MPF
Thalassoma bifasciatum	Labridae	MPF
Thalassoma cupido	Labridae	MPF
Thalassoma duperrey	Labridae	MPF
Thalassoma genivittatum	Labridae	MPF
Thalassoma hardwicke	Labridae	MPF
Thalassoma hebraicum	Labridae	MPF
Thalassoma jansenii	Labridae	MPF
Thalassoma lunare	Labridae	MPF
Thalassoma lutescens	Labridae	MPF
Thalassoma noronhanum	Labridae	MPF
Thalassoma pavo	Labridae	MPF
Thalassoma purpureum	Labridae	MPF
Thalassoma quinquevittatum	Labridae	MPF
Thalassoma trilobatum	Labridae	MPF
Wetmorella nigropinnata	Labridae	MPF
Xiphocheilus typus	Labridae	MPF
Xyrichtys martinicensis	Labridae	MPF
Xyrichtys novacula	Labridae	MPF
Xyrichtys splendens	Labridae	MPF
Alloclinus holderi	Labrisomidae	BCF
Lampris guttatus	Lampridae	MPF
Aurigequula fasciata	Leiognathidae	BCF
Equulites elongatus	Leiognathidae	BCF
Equulites laterofenestra	Leiognathidae	BCF
Equulites leuciscus	Leiognathidae	BCF
Equulites rivulatus	Leiognathidae	BCF
Equulites stercorarius	Leiognathidae	BCF
Eubleekeria jonesi	Leiognathidae	BCF
Eubleekeria splendens	Leiognathidae	BCF
Gazza achlamys	Leiognathidae	BCF
Gazza minuta	Leiognathidae	BCF
Gazza rhombea	Leiognathidae	BCF
Karalla daura	Leiognathidae	BCF
Karalla dussumieri	Leiognathidae	BCF
Leiognathus lineolatus	Leiognathidae	BCF
Leiognathus longispinis	Leiognathidae	BCF
Nuchequula blochii	Leiognathidae	BCF
Nuchequula nuchalis	Leiognathidae	BCF
Secutor indicius	Leiognathidae	BCF
Secutor insidiator	Leiognathidae	BCF
Secutor megalolepis	Leiognathidae	BCF
Gnathodentex aureolineatus	Lethrinidae	BCF
Gymnocranius elongatus	Lethrinidae	BCF
Gymnocranius grandoculis	Lethrinidae	BCF
Gymnocranius griseus	Lethrinidae	BCF
Lethrinus atkinsoni	Lethrinidae	BCF
Lethrinus atlanticus	Lethrinidae	BCF
Lethrinus borbonicus	Lethrinidae	BCF

(continued)

Species	Family	Locomotion Mode
Lethrinus erythracanthus	Lethrinidae	BCF
Lethrinus erythropterus	Lethrinidae	BCF
Lethrinus genivittatus	Lethrinidae	BCF
Lethrinus harak	Lethrinidae	BCF
Lethrinus lentjan	Lethrinidae	BCF
Lethrinus microdon	Lethrinidae	BCF
Lethrinus miniatus	Lethrinidae	BCF
Lethrinus nebulosus	Lethrinidae	BCF
Lethrinus obsoletus	Lethrinidae	BCF
Lethrinus olivaceus	Lethrinidae	BCF
Lethrinus ornatus	Lethrinidae	BCF
Lethrinus reticulatus	Lethrinidae	BCF
Lethrinus rubrioperculatus	Lethrinidae	BCF
Lethrinus semicinctus	Lethrinidae	BCF
Lethrinus xanthochilus	Lethrinidae	BCF
Monotaxis grandoculis	Lethrinidae	BCF
Wattsia mossambica	Lethrinidae	BCF
Careproctus colletti	Liparidae	BCF
Careproctus cypselurus	Liparidae	BCF
Careproctus georgianus	Liparidae	BCF
Careproctus gilberti	Liparidae	BCF
Careproctus melanurus	Liparidae	BCF
Careproctus rastrinus	Liparidae	BCF
Careproctus reinhardti	Liparidae	BCF
Crystallichthys cyclospilus	Liparidae	BCF
Elassodiscus caudatus	Liparidae	BCF
Elassodiscus tremebundus	Liparidae	BCF
Paraliparis dactylosus	Liparidae	BCF
Rhinoliparis attenuatus	Liparidae	BCF
Rhinoliparis barbulifer	Liparidae	BCF
Lobotes pacificus	Lobotidae	BCF
Lobotes surinamensis	Lobotidae	BCF
Brosme brosme	Lotidae	BCF
Ciliata mustela	Lotidae	BCF
Enchelyopus cimbrius	Lotidae	BCF
Gaidropsarus argentatus	Lotidae	BCF
Gaidropsarus ensis	Lotidae	BCF
Gaidropsarus mediterraneus	Lotidae	BCF
Gaidropsarus vulgaris	Lotidae	BCF
Molva macrophthalma	Lotidae	BCF
Aphareus furca	Lutjanidae	BCF
Aprion virescens	Lutjanidae	BCF
Apsilus dentatus	Lutjanidae	BCF
Caesio teres	Lutjanidae	BCF
Lutjanus adetii	Lutjanidae	BCF
Lutjanus analis	Lutjanidae	BCF
Lutjanus apodus	Lutjanidae	BCF
Lutjanus argentiventris	Lutjanidae	BCF
Lutjanus bengalensis	Lutjanidae	BCF
Lutjanus buccanella	Lutjanidae	BCF
Lutjanus campechanus	Lutjanidae	BCF

(continued)

Species	Family	Locomotion Mode
Lutjanus carponotatus	Lutjanidae	BCF
Lutjanus colorado	Lutjanidae	BCF
Lutjanus cyanopterus	Lutjanidae	BCF
Lutjanus decussatus	Lutjanidae	BCF
Lutjanus erythropterus	Lutjanidae	BCF
Lutjanus fulviflamma	Lutjanidae	BCF
Lutjanus gibbus	Lutjanidae	BCF
Lutjanus johnii	Lutjanidae	BCF
Lutjanus kasmira	Lutjanidae	BCF
Lutjanus lutjanus	Lutjanidae	BCF
Lutjanus madras	Lutjanidae	BCF
Lutjanus mahogoni	Lutjanidae	BCF
Lutjanus malabaricus	Lutjanidae	BCF
Lutjanus monostigma	Lutjanidae	BCF
Lutjanus novemfasciatus	Lutjanidae	BCF
Lutjanus peru	Lutjanidae	BCF
Lutjanus purpureus	Lutjanidae	BCF
Lutjanus quinquelineatus	Lutjanidae	BCF
Lutjanus rivulatus	Lutjanidae	BCF
Lutjanus russellii	Lutjanidae	BCF
Lutjanus sanguineus	Lutjanidae	BCF
Lutjanus sebae	Lutjanidae	BCF
Lutjanus synagris	Lutjanidae	BCF
Lutjanus vitta	Lutjanidae	BCF
Lutjanus vivanus	Lutjanidae	BCF
Macolor niger	Lutjanidae	BCF
Ocyurus chrysurus	Lutjanidae	BCF
Paracaesio sordida	Lutjanidae	BCF
Paracaesio xanthura	Lutjanidae	BCF
Pristipomoides aquilonaris	Lutjanidae	BCF
Pristipomoides filamentosus	Lutjanidae	BCF
Pterocaesio tile	Lutjanidae	BCF
Rhomboplites aurorubens	Lutjanidae	BCF
Symphorichthys spilurus	Lutjanidae	BCF
Symphorus nematophorus	Lutjanidae	BCF
Luvarus imperialis	Luvaridae	BCF
Bathygadus antrodes	Macrouridae	BCF
Bathygadus favosus	Macrouridae	BCF
Bathygadus macrops	Macrouridae	BCF
Bathygadus melanobranchus	Macrouridae	BCF
Coelorinchus braueri	Macrouridae	BCF
Coelorinchus caelorhincus	Macrouridae	BCF
Coelorinchus caribbaeus	Macrouridae	BCF
Coelorinchus fasciatus	Macrouridae	BCF
Coelorinchus flabellispinnis	Macrouridae	BCF
Coelorinchus formosanus	Macrouridae	BCF
Coelorinchus japonicus	Macrouridae	BCF
Coelorinchus jordani	Macrouridae	BCF
Coelorinchus kishinouyei	Macrouridae	BCF
Coelorinchus macrochir	Macrouridae	BCF
Coelorinchus marinii	Macrouridae	BCF

(continued)

Species	Family	Locomotion Mode
Coelorinchus occa	Macrouridae	BCF
Coelorinchus oliverianus	Macrouridae	BCF
Coelorinchus scaphopsis	Macrouridae	BCF
Coelorinchus smithi	Macrouridae	BCF
Coryphaenoides acrolepis	Macrouridae	BCF
Coryphaenoides armatus	Macrouridae	BCF
Coryphaenoides carapinus	Macrouridae	BCF
Coryphaenoides cinereus	Macrouridae	BCF
Coryphaenoides leptolepis	Macrouridae	BCF
Coryphaenoides longifilis	Macrouridae	BCF
Coryphaenoides marginatus	Macrouridae	BCF
Coryphaenoides nasutus	Macrouridae	BCF
Coryphaenoides rupestris	Macrouridae	BCF
Coryphaenoides serrulatus	Macrouridae	BCF
Gadomus dispar	Macrouridae	BCF
Hymenocephalus italicus	Macrouridae	BCF
Hymenocephalus longiceps	Macrouridae	BCF
Hymenocephalus striatissimus striatissimus	Macrouridae	BCF
Lucigadus nigromaculatus	Macrouridae	BCF
Macrourus holotrachys	Macrouridae	BCF
Malacocephalus laevis	Macrouridae	BCF
Malacocephalus occidentalis	Macrouridae	BCF
Nezumia aequalis	Macrouridae	BCF
Nezumia bairdii	Macrouridae	BCF
Nezumia liolepis	Macrouridae	BCF
Nezumia longebarbata	Macrouridae	BCF
Nezumia milleri	Macrouridae	BCF
Nezumia proxima	Macrouridae	BCF
Nezumia sclerorhynchus	Macrouridae	BCF
Trachonurus sulcatus	Macrouridae	BCF
Ventrifossa garmani	Macrouridae	BCF
Branchiostegus albus	Malacanthidae	BCF
Branchiostegus argentatus	Malacanthidae	BCF
Branchiostegus auratus	Malacanthidae	BCF
Branchiostegus doliatus	Malacanthidae	BCF
Branchiostegus japonicus	Malacanthidae	BCF
Branchiostegus semifasciatus	Malacanthidae	BCF
Branchiostegus wardi	Malacanthidae	BCF
Caulolatilus affinis	Malacanthidae	BCF
Caulolatilus intermedius	Malacanthidae	BCF
Caulolatilus princeps	Malacanthidae	BCF
Hoplolatilus purpureus	Malacanthidae	BCF
Lopholatilus chamaeleonticeps	Malacanthidae	BCF
Malacanthus brevirostris	Malacanthidae	BCF
Malacanthus latovittatus	Malacanthidae	BCF
Malacanthus plumieri	Malacanthidae	BCF
Melamphaes lugubris	Melamphaidae	BCF
Melamphaes polylepis	Melamphaidae	BCF
Melamphaes simus	Melamphaidae	BCF
Melamphaes suborbitalis	Melamphaidae	BCF
Poromitra capito	Melamphaidae	BCF

(continued)

Species	Family	Locomotion Mode
Poromitra crassiceps	Melamphaidae	BCF
Poromitra megalops	Melamphaidae	BCF
Scopeloberyx robustus	Melamphaidae	BCF
Scopelogadus beanii	Melamphaidae	BCF
Scopelogadus mizolepis bispinosus	Melamphaidae	BCF
Scopelogadus mizolepis mizolepis	Melamphaidae	BCF
Sio nordenskjoldii	Melamphaidae	BCF
Melanocetus johnsonii	Melanocetidae	BCF
Melanocetus murrayi	Melanocetidae	BCF
Melanonus gracilis	Melanonidae	BCF
Melanonus zugmayeri	Melanonidae	BCF
Mene maculata	Menidae	BCF
Merluccius albidus	Merlucciidae	BCF
Merluccius australis	Merlucciidae	BCF
Merluccius bilinearis	Merlucciidae	BCF
Merluccius gayi gayi	Merlucciidae	BCF
Merluccius hubbsi	Merlucciidae	BCF
Merluccius merluccius	Merlucciidae	BCF
Merluccius paradoxus	Merlucciidae	BCF
Merluccius polli	Merlucciidae	BCF
Merluccius productus	Merlucciidae	BCF
Ptereleotris zebra	Microdesmidae	MPF
Nansenia longicauda	Microstomatidae	MPF
Acanthaluteres spilomelanurus	Monacanthidae	MPF
Acanthaluteres vittiger	Monacanthidae	MPF
Acreichthys tomentosus	Monacanthidae	MPF
Aluterus heudelotii	Monacanthidae	BCF
Aluterus monoceros	Monacanthidae	MPF
Aluterus schoepfii	Monacanthidae	MPF
Aluterus scriptus	Monacanthidae	MPF
Amanses scopas	Monacanthidae	MPF
Cantherhines dumerilii	Monacanthidae	MPF
Cantherhines pardalis	Monacanthidae	MPF
Cantherhines pullus	Monacanthidae	MPF
Cantherhines sandwichiensis	Monacanthidae	MPF
Cantherhines verecundus	Monacanthidae	MPF
Chaetodermis penicilligerus	Monacanthidae	MPF
Meuschenia australis	Monacanthidae	MPF
Meuschenia freycineti	Monacanthidae	MPF
Meuschenia hippocrepis	Monacanthidae	MPF
Meuschenia scaber	Monacanthidae	MPF
Meuschenia trachylepis	Monacanthidae	MPF
Monacanthus chinensis	Monacanthidae	MPF
Monacanthus ciliatus	Monacanthidae	MPF
Monacanthus tuckeri	Monacanthidae	MPF
Nelusetta ayraud	Monacanthidae	MPF
Oxymonacanthus longirostris	Monacanthidae	MPF
Paraluteres prionurus	Monacanthidae	MPF
Paramonacanthus choirocephalus	Monacanthidae	MPF
Paramonacanthus oblongus	Monacanthidae	MPF
Paramonacanthus pusillus	Monacanthidae	MPF

(continued)

Species	Family	Locomotion Mode
Paramonacanthus sulcatus	Monacanthidae	MPF
Pervagor janthinosoma	Monacanthidae	MPF
Pervagor melanocephalus	Monacanthidae	MPF
Pervagor nigrolineatus	Monacanthidae	MPF
Pseudalutarius nasicornis	Monacanthidae	MPF
Pseudomonacanthus macrurus	Monacanthidae	MPF
Pseudomonacanthus peroni	Monacanthidae	MPF
Rudarius ercodes	Monacanthidae	MPF
Scobinichthys granulatus	Monacanthidae	MPF
Stephanolepis auratus	Monacanthidae	MPF
Stephanolepis cirrhifer	Monacanthidae	MPF
Stephanolepis hispidus	Monacanthidae	MPF
Stephanolepis setifer	Monacanthidae	MPF
Thamnaconus modestus	Monacanthidae	MPF
Thamnaconus tessellatus	Monacanthidae	MPF
Monocentris japonica	Monocentridae	BCF
Antimora microlepis	Moridae	BCF
Antimora rostrata	Moridae	BCF
Gadella imberbis	Moridae	BCF
Gadella jordani	Moridae	BCF
Halargyreus johnsonii	Moridae	BCF
Laemonema barbatulum	Moridae	BCF
Laemonema goodebeanorum	Moridae	BCF
Lepidion eques	Moridae	BCF
Lepidion inosimae	Moridae	BCF
Mora moro	Moridae	BCF
Physiculus capensis	Moridae	BCF
Physiculus fulvus	Moridae	BCF
Physiculus japonicus	Moridae	BCF
Pseudophycis bachus	Moridae	BCF
Pseudophycis barbata	Moridae	BCF
Tripterophycis gilchristi	Moridae	BCF
Dicentrarchus punctatus	Moronidae	BCF
Liza affinis	Mugilidae	BCF
Liza saliens	Mugilidae	BCF
Mugil bananensis	Mugilidae	BCF
Mugil incilis	Mugilidae	BCF
Oedalechilus labeo	Mugilidae	BCF
Mulloidichthys flavolineatus	Mullidae	BCF
Mulloidichthys martinicus	Mullidae	BCF
Mulloidichthys vanicolensis	Mullidae	BCF
Mullus auratus	Mullidae	BCF
Mullus barbatus barbatus	Mullidae	BCF
Mullus surmuletus	Mullidae	BCF
Parupeneus barberinus	Mullidae	BCF
Parupeneus chrysonemus	Mullidae	BCF
Parupeneus cyclostomus	Mullidae	BCF
Parupeneus forsskali	Mullidae	BCF
Parupeneus heptacanthus	Mullidae	BCF
Parupeneus indicus	Mullidae	BCF
Parupeneus insularis	Mullidae	BCF

(continued)

Species	Family	Locomotion Mode
Parupeneus multifasciatus	Mullidae	BCF
Parupeneus pleurostigma	Mullidae	BCF
Parupeneus spilurus	Mullidae	BCF
Parupeneus trifasciatus	Mullidae	BCF
Pseudupeneus grandisquamis	Mullidae	BCF
Pseudupeneus maculatus	Mullidae	BCF
Upeneichthys lineatus	Mullidae	BCF
Upeneus japonicus	Mullidae	BCF
Upeneus moluccensis	Mullidae	BCF
Upeneus parvus	Mullidae	BCF
Upeneus sulphureus	Mullidae	BCF
Upeneus tragula	Mullidae	BCF
Upeneus vittatus	Mullidae	BCF
Cynoponticus ferox	Muraenesocidae	BCF
Muraenesox bagio	Muraenesocidae	BCF
Oxyconger leptognathus	Muraenesocidae	BCF
Muraenolepis microps	Muraenolepididae	BCF
Muraenolepis orangiensis	Muraenolepididae	BCF
Benthosema fibulatum	Myctophidae	BCF
Benthosema glaciale	Myctophidae	BCF
Benthosema panamense	Myctophidae	BCF
Benthosema pterotum	Myctophidae	BCF
Benthosema suborbitale	Myctophidae	BCF
Bolinichthys distofax	Myctophidae	BCF
Bolinichthys indicus	Myctophidae	BCF
Bolinichthys longipes	Myctophidae	BCF
Bolinichthys supralateralis	Myctophidae	BCF
Centrobranchus nigroocellatus	Myctophidae	BCF
Ceratoscopelus maderensis	Myctophidae	BCF
Ceratoscopelus townsendi	Myctophidae	BCF
Ceratoscopelus warmingii	Myctophidae	BCF
Diaphus anderseni	Myctophidae	BCF
Diaphus bertelseni	Myctophidae	BCF
Diaphus brachycephalus	Myctophidae	BCF
Diaphus dumerilii	Myctophidae	BCF
Diaphus effulgens	Myctophidae	BCF
Diaphus holti	Myctophidae	BCF
Diaphus luetkeni	Myctophidae	BCF
Diaphus metopoclampus	Myctophidae	BCF
Diaphus mollis	Myctophidae	BCF
Diaphus perspicillatus	Myctophidae	BCF
Diaphus rafinesquii	Myctophidae	BCF
Diaphus subtilis	Myctophidae	BCF
Diaphus theta	Myctophidae	BCF
Diaphus thiollierei	Myctophidae	BCF
Diaphus watasei	Myctophidae	BCF
Diogenichthys atlanticus	Myctophidae	BCF
Diogenichthys laternatus	Myctophidae	BCF
Electrona antarctica	Myctophidae	BCF
Electrona carlsbergi	Myctophidae	BCF
Electrona risso	Myctophidae	BCF

(continued)

Species	Family	Locomotion Mode
Gonichthys cocco	Myctophidae	BCF
Gonichthys tenuiculus	Myctophidae	BCF
Gymnoscopelus bolini	Myctophidae	BCF
Gymnoscopelus braueri	Myctophidae	BCF
Gymnoscopelus fraseri	Myctophidae	BCF
Gymnoscopelus nicholsi	Myctophidae	BCF
Gymnoscopelus piabilis	Myctophidae	BCF
Hygophum atratum	Myctophidae	BCF
Hygophum benoiti	Myctophidae	BCF
Hygophum hanseni	Myctophidae	BCF
Hygophum hygomii	Myctophidae	BCF
Hygophum macrochir	Myctophidae	BCF
Hygophum proximum	Myctophidae	BCF
Hygophum reinhardtii	Myctophidae	BCF
Hygophum taaningi	Myctophidae	BCF
Lampadena anomala	Myctophidae	BCF
Lampadena chavesi	Myctophidae	BCF
Lampadena speculigera	Myctophidae	BCF
Lampadena urophaos urophaos	Myctophidae	BCF
Lampanyctus alatus	Myctophidae	BCF
Lampanyctus australis	Myctophidae	BCF
Lampanyctus crocodilus	Myctophidae	BCF
Lampanyctus intricarius	Myctophidae	BCF
Lampanyctus macdonaldi	Myctophidae	BCF
Lampanyctus nobilis	Myctophidae	BCF
Lampanyctus photonotus	Myctophidae	BCF
Lampanyctus pusillus	Myctophidae	BCF
Lampanyctus tenuiformis	Myctophidae	BCF
Lampichthys procerus	Myctophidae	BCF
Lepidophanes guentheri	Myctophidae	BCF
Lobianchia dofleini	Myctophidae	BCF
Lobianchia gemellarii	Myctophidae	BCF
Metelectrona ventralis	Myctophidae	BCF
Myctophum asperum	Myctophidae	BCF
Myctophum aurolaternatum	Myctophidae	BCF
Myctophum lychnobium	Myctophidae	BCF
Myctophum nitidulum	Myctophidae	BCF
Myctophum punctatum	Myctophidae	BCF
Myctophum spinosum	Myctophidae	BCF
Nannobrachium atrum	Myctophidae	BCF
Nannobrachium cuprarium	Myctophidae	BCF
Nannobrachium lineatum	Myctophidae	BCF
Nannobrachium ritteri	Myctophidae	BCF
Notoscopelus caudispinosus	Myctophidae	BCF
Notoscopelus elongatus	Myctophidae	BCF
Notoscopelus resplendens	Myctophidae	BCF
Parvilux ingens	Myctophidae	BCF
Protomyctophum andriashevi	Myctophidae	BCF
Protomyctophum arcticum	Myctophidae	BCF
Protomyctophum bolini	Myctophidae	BCF
Protomyctophum crockeri	Myctophidae	BCF

(continued)

Species	Family	Locomotion Mode
Protomyctophum gemmatum	Myctophidae	BCF
Protomyctophum parallelum	Myctophidae	BCF
Scopelopsis multipunctatus	Myctophidae	BCF
Stenobrachius leucopsarus	Myctophidae	BCF
Stenobrachius nannochir	Myctophidae	BCF
Symbolophorus barnardi	Myctophidae	BCF
Symbolophorus boops	Myctophidae	BCF
Symbolophorus californiensis	Myctophidae	BCF
Symbolophorus evermanni	Myctophidae	BCF
Symbolophorus veranyi	Myctophidae	BCF
Taaningichthys bathyphilus	Myctophidae	BCF
Tarletonbeania crenularis	Myctophidae	BCF
Triphoturus nigrescens	Myctophidae	BCF
Avocettina infans	Nemichthyidae	BCF
Nemichthys curvirostris	Nemichthyidae	BCF
Nemichthys scolopaceus	Nemichthyidae	BCF
Nemipterus bathybius	Nemipteridae	BCF
Nemipterus bipunctatus	Nemipteridae	BCF
Nemipterus furcosus	Nemipteridae	BCF
Nemipterus japonicus	Nemipteridae	BCF
Nemipterus marginatus	Nemipteridae	BCF
Nemipterus nematophorus	Nemipteridae	BCF
Nemipterus peronii	Nemipteridae	BCF
Nemipterus virgatus	Nemipteridae	BCF
Nemipterus zysron	Nemipteridae	BCF
Parascolopsis eriomma	Nemipteridae	BCF
Pentapodus aureofasciatus	Nemipteridae	BCF
Pentapodus bifasciatus	Nemipteridae	BCF
Pentapodus caninus	Nemipteridae	BCF
Pentapodus nagasakiensis	Nemipteridae	BCF
Pentapodus setosus	Nemipteridae	BCF
Scolopsis affinis	Nemipteridae	BCF
Scolopsis bilineata	Nemipteridae	BCF
Scolopsis bimaculata	Nemipteridae	BCF
Scolopsis ciliata	Nemipteridae	BCF
Scolopsis margaritifera	Nemipteridae	BCF
Scolopsis monogramma	Nemipteridae	BCF
Scolopsis taenioptera	Nemipteridae	BCF
Scolopsis vosmeri	Nemipteridae	BCF
Neoscopelus macrolepidotus	Neoscopelidae	BCF
Neoscopelus microchir	Neoscopelidae	BCF
Scopelengys tristis	Neoscopelidae	BCF
Neosebastes thetidis	Neosebastidae	BCF
Hoplunnis tenuis	Nettastomatidae	BCF
Nettastoma melanurum	Nettastomatidae	BCF
Saurenchelys fierasfer	Nettastomatidae	BCF
Venefica procera	Nettastomatidae	BCF
Cubiceps baxteri	Nomeidae	BCF
Cubiceps capensis	Nomeidae	BCF
Cubiceps gracilis	Nomeidae	BCF
Cubiceps pauciradiatus	Nomeidae	BCF

(continued)

Species	Family	Locomotion Mode
Cubiceps whiteleggii	Nomeidae	BCF
Psenes arafurensis	Nomeidae	MPF
Psenes cyanophrys	Nomeidae	MPF
Psenes pellucidus	Nomeidae	MPF
Normanichthys crockeri	Normanichthyidae	BCF
Notacanthus abbotti	Notacanthidae	BCF
Notacanthus bonaparte	Notacanthidae	BCF
Polyacanthonotus challengeri	Notacanthidae	BCF
Polyacanthonotus rissoanus	Notacanthidae	BCF
Ahliesaurus berryi	Notosudidae	BCF
Scopelosaurus harryi	Notosudidae	BCF
Scopelosaurus lepidus	Notosudidae	BCF
Dissostichus eleginoides	Nototheniidae	MPF
Dissostichus mawsoni	Nototheniidae	MPF
Lepidonotothen larseni	Nototheniidae	MPF
Pagothenia borchgrevinki	Nototheniidae	MPF
Paranotothenia magellanica	Nototheniidae	MPF
Pleuragramma antarctica	Nototheniidae	MPF
Haletta semifasciata	Odacidae	MPF
Neoodax balteatus	Odacidae	MPF
Olisthops cyanomelas	Odacidae	MPF
Omosudis lowii	Omosudidae	BCF
Oneirodes acanthias	Oneirodidae	BCF
Bassozetus zenkevitchi	Ophidiidae	BCF
Brotula barbata	Ophidiidae	BCF
Brotula multibarbata	Ophidiidae	BCF
Brotulotaenia crassa	Ophidiidae	BCF
Brotulotaenia nigra	Ophidiidae	BCF
Dicrolene introniger	Ophidiidae	BCF
Genypterus blacodes	Ophidiidae	BCF
Genypterus capensis	Ophidiidae	BCF
Lamprogrammus niger	Ophidiidae	BCF
Lepophidium brevibarbe	Ophidiidae	BCF
Lepophidium jeannae	Ophidiidae	BCF
Lepophidium profundorum	Ophidiidae	BCF
Neobythites gilli	Ophidiidae	BCF
Neobythites sivicola	Ophidiidae	BCF
Neobythites stigmosus	Ophidiidae	BCF
Ophidion holbrookii	Ophidiidae	BCF
Ophidion josephi	Ophidiidae	BCF
Petrotyx sanguineus	Ophidiidae	BCF
Selachophidium guentheri	Ophidiidae	BCF
Sirembo imberbis	Ophidiidae	BCF
Macropinna microstoma	Opisthoproctidae	BCF
Rhynchohyalus natalensis	Opisthoproctidae	BCF
Oplegnathus fasciatus	Oplegnathidae	BCF
Oplegnathus punctatus	Oplegnathidae	BCF
Allocyttus verrucosus	Oreosomatidae	MPF
Allosmerus elongatus	Osmeridae	BCF
Hypomesus pretiosus	Osmeridae	BCF
Spirinchus starksi	Osmeridae	BCF

(continued)

Species	Family	Locomotion Mode
Acanthostracion polygonius	Ostraciidae	MPF
Acanthostracion quadricornis	Ostraciidae	MPF
Lactophrys bicaudalis	Ostraciidae	MPF
Lactophrys trigonus	Ostraciidae	MPF
Lactoria cornuta	Ostraciidae	MPF
Lactoria diaphana	Ostraciidae	MPF
Ostracion immaculatus	Ostraciidae	MPF
Ostracion meleagris	Ostraciidae	MPF
Ostracion rhinorhynchos	Ostraciidae	MPF
Ostracion solorensis	Ostraciidae	MPF
Ostracion whitleyi	Ostraciidae	MPF
Rhinesomus triqueter	Ostraciidae	MPF
Tetrosomus concatenatus	Ostraciidae	MPF
Tetrosomus gibbosus	Ostraciidae	MPF
Lestidiops jayakari jayakari	Paralepididae	BCF
Lestidium atlanticum	Paralepididae	BCF
Lestrolepis intermedia	Paralepididae	BCF
Magnisudis atlantica	Paralepididae	BCF
Notolepis coatsi	Paralepididae	BCF
Cyttopsis cypho	Parazenidae	MPF
Cyttopsis rosea	Parazenidae	MPF
Parapriacanthus ransonneti	Pempheridae	MPF
Pempheris mangula	Pempheridae	MPF
Pempheris oualensis	Pempheridae	MPF
Pempheris schomburgkii	Pempheridae	MPF
Pempheris schwenkii	Pempheridae	MPF
Pempheris vanicolensis	Pempheridae	MPF
Histiopterus typus	Pentacerotidae	MPF
Ichthyococcus ovatus	Phosichthyidae	BCF
Phosichthys argenteus	Phosichthyidae	BCF
Polymetme corythaeola	Phosichthyidae	BCF
Polymetme thaeocoryla	Phosichthyidae	BCF
Yarrella blackfordi	Phosichthyidae	BCF
Phycis blennoides	Phycidae	BCF
Phycis chesteri	Phycidae	BCF
Phycis phycis	Phycidae	BCF
Urophycis brasiliensis	Phycidae	BCF
Urophycis chuss	Phycidae	BCF
Urophycis cirrata	Phycidae	BCF
Urophycis floridana	Phycidae	BCF
Holtbyrnia latifrons	Platytroctidae	BCF
Maulisia microlepis	Platytroctidae	BCF
Normichthys operosus	Platytroctidae	BCF
Sagamichthys abei	Platytroctidae	BCF
Searsia koefoedi	Platytroctidae	BCF
Cnidoglanis macrocephalus	Plotosidae	BCF
Plotosus lineatus	Plotosidae	BCF
Polymixia berndti	Polymixiidae	BCF
Polymixia japonica	Polymixiidae	BCF
Polymixia lowei	Polymixiidae	BCF
Polymixia nobilis	Polymixiidae	BCF

(continued)

Species	Family	Locomotion Mode
Pentanemus quinquarius	Polynemidae	BCF
Polydactylus approximans	Polynemidae	BCF
Polydactylus plebeius	Polynemidae	BCF
Polyprion americanus	Polyprionidae	BCF
Stereolepis gigas	Polyprionidae	BCF
Apolemichthys trimaculatus	Pomacanthidae	MPF
Apolemichthys xanthurus	Pomacanthidae	MPF
Centropyge aurantia	Pomacanthidae	MPF
Centropyge aurantonotus	Pomacanthidae	MPF
Centropyge bicolor	Pomacanthidae	MPF
Centropyge bispinosa	Pomacanthidae	MPF
Centropyge eibli	Pomacanthidae	MPF
Centropyge ferrugata	Pomacanthidae	MPF
Centropyge flavicauda	Pomacanthidae	MPF
Centropyge flavissima	Pomacanthidae	MPF
Centropyge loriculus	Pomacanthidae	MPF
Centropyge multispinis	Pomacanthidae	MPF
Centropyge nox	Pomacanthidae	MPF
Centropyge potteri	Pomacanthidae	MPF
Centropyge tibicen	Pomacanthidae	MPF
Centropyge venusta	Pomacanthidae	MPF
Centropyge vrolikii	Pomacanthidae	MPF
Chaetodontoplus duboulayi	Pomacanthidae	MPF
Chaetodontoplus melanosoma	Pomacanthidae	MPF
Chaetodontoplus mesoleucus	Pomacanthidae	MPF
Genicanthus lamarck	Pomacanthidae	MPF
Genicanthus melanospilos	Pomacanthidae	MPF
Holacanthus bermudensis	Pomacanthidae	MPF
Holacanthus ciliaris	Pomacanthidae	MPF
Holacanthus limbaughi	Pomacanthidae	MPF
Holacanthus passer	Pomacanthidae	MPF
Holacanthus tricolor	Pomacanthidae	MPF
Paracentropyge multifasciata	Pomacanthidae	MPF
Pomacanthus annularis	Pomacanthidae	MPF
Pomacanthus arcuatus	Pomacanthidae	MPF
Pomacanthus asfur	Pomacanthidae	MPF
Pomacanthus chrysurus	Pomacanthidae	MPF
Pomacanthus imperator	Pomacanthidae	MPF
Pomacanthus maculosus	Pomacanthidae	MPF
Pomacanthus navarchus	Pomacanthidae	MPF
Pomacanthus paru	Pomacanthidae	MPF
Pomacanthus semicirculatus	Pomacanthidae	MPF
Pomacanthus sexstriatus	Pomacanthidae	MPF
Pomacanthus xanthometopon	Pomacanthidae	MPF
Pomacanthus zonipectus	Pomacanthidae	MPF
Pygoplites diacanthus	Pomacanthidae	MPF
Abudefduf abdominalis	Pomacentridae	MPF
Abudefduf bengalensis	Pomacentridae	MPF
Abudefduf concolor	Pomacentridae	MPF
Abudefduf declivifrons	Pomacentridae	MPF
Abudefduf lorenzi	Pomacentridae	MPF

(continued)

Species	Family	Locomotion Mode
Abudefduf luridus	Pomacentridae	MPF
Abudefduf margariteus	Pomacentridae	MPF
Abudefduf notatus	Pomacentridae	MPF
Abudefduf saxatilis	Pomacentridae	MPF
Abudefduf septemfasciatus	Pomacentridae	MPF
Abudefduf sexfasciatus	Pomacentridae	MPF
Abudefduf sordidus	Pomacentridae	MPF
Abudefduf sparoides	Pomacentridae	MPF
Abudefduf taurus	Pomacentridae	MPF
Abudefduf troschelii	Pomacentridae	MPF
Abudefduf vaigiensis	Pomacentridae	MPF
Abudefduf whitleyi	Pomacentridae	MPF
Acanthochromis polyacanthus	Pomacentridae	MPF
Altrichthys curatus	Pomacentridae	MPF
Amblyglyphidodon aureus	Pomacentridae	MPF
Amblyglyphidodon curacao	Pomacentridae	MPF
Amblyglyphidodon leucogaster	Pomacentridae	MPF
Cheiloprion labiatus	Pomacentridae	MPF
Chromis agilis	Pomacentridae	MPF
Chromis alpha	Pomacentridae	MPF
Chromis alta	Pomacentridae	MPF
Chromis amboinensis	Pomacentridae	MPF
Chromis analis	Pomacentridae	MPF
Chromis atrilobata	Pomacentridae	BCF
Chromis atripectoralis	Pomacentridae	MPF
Chromis atripes	Pomacentridae	MPF
Chromis caudalis	Pomacentridae	MPF
Chromis chromis	Pomacentridae	MPF
Chromis chrysura	Pomacentridae	MPF
Chromis cyanea	Pomacentridae	MPF
Chromis iomelas	Pomacentridae	MPF
Chromis limbata	Pomacentridae	MPF
Chromis margaritifer	Pomacentridae	MPF
Chromis multilineata	Pomacentridae	MPF
Chromis notata	Pomacentridae	MPF
Chromis opercularis	Pomacentridae	MPF
Chromis ovatiformes	Pomacentridae	MPF
Chromis punctipinnis	Pomacentridae	MPF
Chromis retrofasciata	Pomacentridae	MPF
Chromis ternatensis	Pomacentridae	MPF
Chromis weberi	Pomacentridae	MPF
Chromis xanthochira	Pomacentridae	MPF
Chromis xanthopterygia	Pomacentridae	MPF
Chromis xanthura	Pomacentridae	MPF
Chrysiptera annulata	Pomacentridae	MPF
Chrysiptera brownriggii	Pomacentridae	MPF
Chrysiptera cyanea	Pomacentridae	MPF
Chrysiptera glauca	Pomacentridae	MPF
Chrysiptera hemicyanea	Pomacentridae	MPF
Chrysiptera oxycephala	Pomacentridae	MPF
Chrysiptera rex	Pomacentridae	MPF

(continued)

Species	Family	Locomotion Mode
Chrysiptera rollandi	Pomacentridae	MPF
Chrysiptera springeri	Pomacentridae	MPF
Chrysiptera talboti	Pomacentridae	MPF
Chrysiptera taupou	Pomacentridae	MPF
Chrysiptera unimaculata	Pomacentridae	MPF
Dascyllus albisella	Pomacentridae	MPF
Dascyllus aruanus	Pomacentridae	MPF
Dascyllus carneus	Pomacentridae	MPF
Dascyllus flavicaudus	Pomacentridae	MPF
Dascyllus marginatus	Pomacentridae	MPF
Dascyllus melanurus	Pomacentridae	MPF
Dascyllus reticulatus	Pomacentridae	MPF
Dascyllus trimaculatus	Pomacentridae	MPF
Dischistodus chrysopoecilus	Pomacentridae	MPF
Dischistodus melanotus	Pomacentridae	MPF
Dischistodus perspicillatus	Pomacentridae	MPF
Dischistodus prosopotaenia	Pomacentridae	MPF
Dischistodus pseudochrysopoecilus	Pomacentridae	MPF
Hemiglyphidodon plagiometopon	Pomacentridae	MPF
Hypsypops rubicundus	Pomacentridae	MPF
Lepidozygus tapeinosoma	Pomacentridae	BCF
Mecaenichthys immaculatus	Pomacentridae	MPF
Microspathodon chrysurus	Pomacentridae	MPF
Microspathodon dorsalis	Pomacentridae	MPF
Neoglyphidodon melas	Pomacentridae	MPF
Neoglyphidodon nigroris	Pomacentridae	MPF
Neoglyphidodon oxyodon	Pomacentridae	MPF
Neoglyphidodon polyacanthus	Pomacentridae	MPF
Neoglyphidodon thoracotaeniatus	Pomacentridae	MPF
Neopomacentrus azysron	Pomacentridae	MPF
Neopomacentrus cyanomos	Pomacentridae	MPF
Neopomacentrus filamentosus	Pomacentridae	MPF
Neopomacentrus miryae	Pomacentridae	MPF
Neopomacentrus nemurus	Pomacentridae	MPF
Neopomacentrus sindensis	Pomacentridae	MPF
Parma microlepis	Pomacentridae	MPF
Plectroglyphidodon dickii	Pomacentridae	MPF
Plectroglyphidodon lacrymatus	Pomacentridae	MPF
Plectroglyphidodon leucozonus	Pomacentridae	MPF
Pomacentrus adelus	Pomacentridae	MPF
Pomacentrus alexanderae	Pomacentridae	MPF
Pomacentrus amboinensis	Pomacentridae	MPF
Pomacentrus auriventris	Pomacentridae	MPF
Pomacentrus bankanensis	Pomacentridae	MPF
Pomacentrus brachialis	Pomacentridae	MPF
Pomacentrus burroughi	Pomacentridae	MPF
Pomacentrus chrysurus	Pomacentridae	MPF
Pomacentrus coelestis	Pomacentridae	MPF
Pomacentrus grammorhynchus	Pomacentridae	MPF
Pomacentrus lepidogenys	Pomacentridae	MPF
Pomacentrus moluccensis	Pomacentridae	MPF

(continued)

Species	Family	Locomotion Mode
Pomacentrus nagasakiensis	Pomacentridae	MPF
Pomacentrus nigromanus	Pomacentridae	MPF
Pomacentrus pavo	Pomacentridae	MPF
Pomacentrus philippinus	Pomacentridae	MPF
Pomacentrus reidi	Pomacentridae	MPF
Pomacentrus smithi	Pomacentridae	MPF
Pomacentrus trilineatus	Pomacentridae	MPF
Pomacentrus vaiuli	Pomacentridae	MPF
Pomachromis richardsoni	Pomacentridae	MPF
Premnas biaculeatus	Pomacentridae	MPF
Pristotis obtusirostris	Pomacentridae	BCF
Stegastes adustus	Pomacentridae	MPF
Stegastes altus	Pomacentridae	MPF
Stegastes apicalis	Pomacentridae	MPF
Stegastes diencaeus	Pomacentridae	MPF
Stegastes fasciolatus	Pomacentridae	MPF
Stegastes imbricatus	Pomacentridae	MPF
Stegastes leucostictus	Pomacentridae	MPF
Stegastes lividus	Pomacentridae	MPF
Stegastes nigricans	Pomacentridae	MPF
Stegastes obreptus	Pomacentridae	MPF
Stegastes partitus	Pomacentridae	MPF
Stegastes planifrons	Pomacentridae	MPF
Stegastes variabilis	Pomacentridae	MPF
Teixeirichthys jordani	Pomacentridae	BCF
Pomatomus saltatrix	Pomatomidae	BCF
Cookeolus japonicus	Priacanthidae	BCF
Heteropriacanthus cruentatus	Priacanthidae	BCF
Priacanthus arenatus	Priacanthidae	BCF
Priacanthus hamrur	Priacanthidae	BCF
Priacanthus tayenus	Priacanthidae	BCF
Pristigenys alta	Priacanthidae	MPF
Pristigenys serrula	Priacanthidae	MPF
Ilisha elongata	Pristigasteridae	BCF
Ilisha melastoma	Pristigasteridae	BCF
Ilisha striatula	Pristigasteridae	BCF
Opisthopterus tardoore	Pristigasteridae	BCF
Pellona harroweri	Pristigasteridae	BCF
Pseudochromis bitaeniatus	Pseudochromidae	MPF
Rachycentron canadum	Rachycentridae	BCF
Rondeletia bicolor	Rondeletiidae	BCF
Rondeletia loricata	Rondeletiidae	BCF
Bolbometopon muricatum	Scaridae	MPF
Calotomus carolinus	Scaridae	MPF
Calotomus spinidens	Scaridae	MPF
Cetoscarus bicolor	Scaridae	MPF
Chlorurus bleekeri	Scaridae	MPF
Chlorurus bowersi	Scaridae	MPF
Chlorurus capistratoides	Scaridae	MPF
Chlorurus gibbus	Scaridae	MPF
Chlorurus japanensis	Scaridae	MPF

(continued)

Species	Family	Locomotion Mode
Chlorurus microrhinos	Scaridae	MPF
Chlorurus oedema	Scaridae	MPF
Chlorurus sordidus	Scaridae	MPF
Cryptotomus roseus	Scaridae	MPF
Hipposcarus harid	Scaridae	MPF
Hipposcarus longiceps	Scaridae	MPF
Leptoscarus vaigiensis	Scaridae	MPF
Nicholsina denticulata	Scaridae	MPF
Nicholsina usta usta	Scaridae	MPF
Scarus altipinnis	Scaridae	MPF
Scarus chameleon	Scaridae	MPF
Scarus coelestinus	Scaridae	MPF
Scarus dimidiatus	Scaridae	MPF
Scarus festivus	Scaridae	MPF
Scarus flavipectoralis	Scaridae	MPF
Scarus forsteni	Scaridae	MPF
Scarus frenatus	Scaridae	MPF
Scarus ghobban	Scaridae	MPF
Scarus globiceps	Scaridae	MPF
Scarus guacamaia	Scaridae	MPF
Scarus hypselopterus	Scaridae	MPF
Scarus iseri	Scaridae	MPF
Scarus niger	Scaridae	MPF
Scarus oviceps	Scaridae	MPF
Scarus prasiognathos	Scaridae	MPF
Scarus psittacus	Scaridae	MPF
Scarus quoyi	Scaridae	MPF
Scarus rubroviolaceus	Scaridae	MPF
Scarus schlegeli	Scaridae	MPF
Scarus taeniopterus	Scaridae	MPF
Scarus tricolor	Scaridae	MPF
Sparisoma atomarium	Scaridae	MPF
Sparisoma aurofrenatum	Scaridae	MPF
Sparisoma chrysopterum	Scaridae	MPF
Sparisoma cretense	Scaridae	MPF
Sparisoma frondosum	Scaridae	MPF
Sparisoma radians	Scaridae	MPF
Sparisoma rubripinne	Scaridae	MPF
Sparisoma strigatum	Scaridae	MPF
Sparisoma viride	Scaridae	MPF
Atractoscion aequidens	Sciaenidae	BCF
Atrobucca nibe	Sciaenidae	BCF
Bairdiella armata	Sciaenidae	BCF
Bairdiella ronchus	Sciaenidae	BCF
Cheilotrema saturnum	Sciaenidae	BCF
Collichthys lucidus	Sciaenidae	BCF
Cynoscion albus	Sciaenidae	BCF
Cynoscion analis	Sciaenidae	BCF
Cynoscion arenarius	Sciaenidae	BCF
Cynoscion jamaicensis	Sciaenidae	BCF
Cynoscion leiarchus	Sciaenidae	BCF

(continued)

Species	Family	Locomotion Mode
Cynoscion microlepidotus	Sciaenidae	BCF
Cynoscion nebulosus	Sciaenidae	BCF
Cynoscion nothus	Sciaenidae	BCF
Cynoscion othonopterus	Sciaenidae	BCF
Cynoscion phoxocephalus	Sciaenidae	BCF
Cynoscion regalis	Sciaenidae	BCF
Cynoscion reticulatus	Sciaenidae	BCF
Cynoscion squamipinnis	Sciaenidae	BCF
Cynoscion virescens	Sciaenidae	BCF
Equetus punctatus	Sciaenidae	BCF
Genyonemus lineatus	Sciaenidae	BCF
Isopisthus remifer	Sciaenidae	BCF
Johnius belangerii	Sciaenidae	BCF
Johnius dussumieri	Sciaenidae	BCF
Larimichthys crocea	Sciaenidae	BCF
Larimichthys polyactis	Sciaenidae	BCF
Leiostomus xanthurus	Sciaenidae	BCF
Lonchurus lanceolatus	Sciaenidae	BCF
Macrodon ancylodon	Sciaenidae	BCF
Macrodon mordax	Sciaenidae	BCF
Menticirrhus americanus	Sciaenidae	BCF
Menticirrhus littoralis	Sciaenidae	BCF
Menticirrhus undulatus	Sciaenidae	BCF
Micropogonias furnieri	Sciaenidae	BCF
Micropogonias undulatus	Sciaenidae	BCF
Miichthys miiuy	Sciaenidae	BCF
Nebris microps	Sciaenidae	BCF
Nebris occidentalis	Sciaenidae	BCF
Nibea albiflora	Sciaenidae	BCF
Otolithes cuvieri	Sciaenidae	BCF
Otolithes ruber	Sciaenidae	BCF
Otolithoides biauritus	Sciaenidae	BCF
Pareques umbrosus	Sciaenidae	BCF
Pennahia anea	Sciaenidae	BCF
Pennahia argentata	Sciaenidae	BCF
Pogonias cromis	Sciaenidae	BCF
Protonibea diacanthus	Sciaenidae	BCF
Pseudotolithus senegallus	Sciaenidae	BCF
Roncador stearnsii	Sciaenidae	BCF
Seriphus politus	Sciaenidae	BCF
Stellifer illecebrosus	Sciaenidae	BCF
Stellifer lanceolatus	Sciaenidae	BCF
Stellifer microps	Sciaenidae	BCF
Stellifer naso	Sciaenidae	BCF
Stellifer rastrifer	Sciaenidae	BCF
Umbrina canariensis	Sciaenidae	BCF
Umbrina roncador	Sciaenidae	BCF
Cololabis adocetus	Scomberesocidae	BCF
Cololabis saira	Scomberesocidae	BCF
Acanthocybium solandri	Scombridae	BCF
Auxis rochei rochei	Scombridae	BCF

(continued)

Species	Family	Locomotion Mode
Euthynnus affinis	Scombridae	BCF
Euthynnus alletteratus	Scombridae	BCF
Euthynnus lineatus	Scombridae	BCF
Gasterochisma melampus	Scombridae	BCF
Grammatorcynus bilineatus	Scombridae	BCF
Gymnosarda unicolor	Scombridae	BCF
Katsuwonus pelamis	Scombridae	BCF
Rastrelliger brachysoma	Scombridae	BCF
Rastrelliger faughni	Scombridae	BCF
Sarda chiliensis chiliensis	Scombridae	BCF
Sarda orientalis	Scombridae	BCF
Sarda sarda	Scombridae	BCF
Scomber australasicus	Scombridae	BCF
Scomber colias	Scombridae	BCF
Scomber japonicus	Scombridae	BCF
Scomber scombrus	Scombridae	BCF
Scomberomorus brasiliensis	Scombridae	BCF
Scomberomorus cavalla	Scombridae	BCF
Scomberomorus commerson	Scombridae	BCF
Scomberomorus concolor	Scombridae	BCF
Scomberomorus guttatus	Scombridae	BCF
Scomberomorus koreanus	Scombridae	BCF
Scomberomorus maculatus	Scombridae	BCF
Scomberomorus plurilineatus	Scombridae	BCF
Scomberomorus queenslandicus	Scombridae	BCF
Scomberomorus regalis	Scombridae	BCF
Scomberomorus sierra	Scombridae	BCF
Thunnus albacares	Scombridae	BCF
Thunnus atlanticus	Scombridae	BCF
Thunnus obesus	Scombridae	BCF
Thunnus thynnus	Scombridae	BCF
Thunnus tonggol	Scombridae	BCF
Benthalbella elongata	Scopelarchidae	BCF
Benthalbella infans	Scopelarchidae	BCF
Benthalbella macropinna	Scopelarchidae	BCF
Dendrochirus biocellatus	Scorpaenidae	BCF
Dendrochirus brachypterus	Scorpaenidae	BCF
Dendrochirus zebra	Scorpaenidae	BCF
Neomerinthe hemingwayi	Scorpaenidae	BCF
Neomerinthe rotunda	Scorpaenidae	BCF
Pontinus kuhlii	Scorpaenidae	BCF
Pontinus longispinis	Scorpaenidae	BCF
Pontinus rathbuni	Scorpaenidae	BCF
Pterois andover	Scorpaenidae	BCF
Pterois antennata	Scorpaenidae	BCF
Pterois miles	Scorpaenidae	BCF
Pterois radiata	Scorpaenidae	BCF
Pterois russelii	Scorpaenidae	BCF
Pterois volitans	Scorpaenidae	BCF
Helicolenus avius	Sebastidae	BCF
Helicolenus dactylopterus	Sebastidae	BCF

(continued)

Species	Family	Locomotion Mode
Helicolenus hilgendorfii	Sebastidae	BCF
Helicolenus lengerichi	Sebastidae	BCF
Sebastes aleutianus	Sebastidae	BCF
Sebastes alutus	Sebastidae	BCF
Sebastes auriculatus	Sebastidae	BCF
Sebastes aurora	Sebastidae	BCF
Sebastes brevispinis	Sebastidae	BCF
Sebastes capensis	Sebastidae	BCF
Sebastes caurinus	Sebastidae	BCF
Sebastes ciliatus	Sebastidae	BCF
Sebastes crameri	Sebastidae	BCF
Sebastes diploproa	Sebastidae	BCF
Sebastes emphaeus	Sebastidae	BCF
Sebastes ensifer	Sebastidae	BCF
Sebastes eos	Sebastidae	BCF
Sebastes fasciatus	Sebastidae	BCF
Sebastes flammeus	Sebastidae	BCF
Sebastes flavidus	Sebastidae	BCF
Sebastes glaucus	Sebastidae	BCF
Sebastes hubbsi	Sebastidae	BCF
Sebastes inermis	Sebastidae	BCF
Sebastes iracundus	Sebastidae	BCF
Sebastes jordani	Sebastidae	BCF
Sebastes joyneri	Sebastidae	BCF
Sebastes maliger	Sebastidae	BCF
Sebastes melanops	Sebastidae	BCF
Sebastes melanostomus	Sebastidae	BCF
Sebastes miniatus	Sebastidae	BCF
Sebastes mystinus	Sebastidae	BCF
Sebastes nigrocinctus	Sebastidae	BCF
Sebastes nivosus	Sebastidae	BCF
Sebastes norvegicus	Sebastidae	BCF
Sebastes oblongus	Sebastidae	BCF
Sebastes ovalis	Sebastidae	BCF
Sebastes owstoni	Sebastidae	BCF
Sebastes pachycephalus	Sebastidae	BCF
Sebastes paucispinis	Sebastidae	BCF
Sebastes pinniger	Sebastidae	BCF
Sebastes polyspinis	Sebastidae	BCF
Sebastes proriger	Sebastidae	BCF
Sebastes reedi	Sebastidae	BCF
Sebastes ruberrimus	Sebastidae	BCF
Sebastes saxicola	Sebastidae	BCF
Sebastes schlegelii	Sebastidae	BCF
Sebastes semicinctus	Sebastidae	BCF
Sebastes serriceps	Sebastidae	BCF
Sebastes steindachneri	Sebastidae	BCF
Sebastes taczanowskii	Sebastidae	BCF
Sebastes thompsoni	Sebastidae	BCF
Sebastes trivittatus	Sebastidae	BCF
Sebastes umbrosus	Sebastidae	BCF

(continued)

Species	Family	Locomotion Mode
Sebastes variabilis	Sebastidae	BCF
Sebastes variegatus	Sebastidae	BCF
Sebastes viviparus	Sebastidae	BCF
Sebastes vulpes	Sebastidae	BCF
Sebastes zacentrus	Sebastidae	BCF
Aethaloperca rogaa	Serranidae	BCF
Anthias anthias	Serranidae	BCF
Anyperodon leucogrammicus	Serranidae	BCF
Baldwinella vivanus	Serranidae	BCF
Belonoperca chabanaudi	Serranidae	BCF
Cromileptes altivelis	Serranidae	BCF
Dermatolepis inermis	Serranidae	BCF
Diplectrum formosum	Serranidae	BCF
Diplectrum maximum	Serranidae	BCF
Diplectrum pacificum	Serranidae	BCF
Diploprion bifasciatum	Serranidae	BCF
Epinephelus aeneus	Serranidae	BCF
Epinephelus akaara	Serranidae	BCF
Epinephelus amblycephalus	Serranidae	BCF
Epinephelus analogus	Serranidae	BCF
Epinephelus areolatus	Serranidae	BCF
Epinephelus chlorostigma	Serranidae	BCF
Epinephelus clippertonensis	Serranidae	BCF
Epinephelus costae	Serranidae	BCF
Epinephelus cyanopodus	Serranidae	BCF
Epinephelus daemelii	Serranidae	BCF
Epinephelus diacanthus	Serranidae	BCF
Epinephelus epistictus	Serranidae	BCF
Epinephelus fasciatomaculosus	Serranidae	BCF
Epinephelus flavocaeruleus	Serranidae	BCF
Epinephelus fuscoguttatus	Serranidae	BCF
Epinephelus itajara	Serranidae	BCF
Epinephelus latifasciatus	Serranidae	BCF
Epinephelus longispinis	Serranidae	BCF
Epinephelus marginatus	Serranidae	BCF
Epinephelus morrhua	Serranidae	BCF
Epinephelus multinotatus	Serranidae	BCF
Epinephelus radiatus	Serranidae	BCF
Epinephelus retouti	Serranidae	BCF
Epinephelus sexfasciatus	Serranidae	BCF
Epinephelus striatus	Serranidae	BCF
Epinephelus undulosus	Serranidae	BCF
Gracila albomarginata	Serranidae	BCF
Grammistes sexlineatus	Serranidae	BCF
Grammistops ocellatus	Serranidae	BCF
Hemanthias leptus	Serranidae	BCF
Hemanthias signifer	Serranidae	BCF
Hypoplectrus aberrans	Serranidae	BCF
Hypoplectrus chlorurus	Serranidae	BCF
Hypoplectrus guttavarius	Serranidae	BCF
Hypoplectrus indigo	Serranidae	BCF

(continued)

Species	Family	Locomotion Mode
Hypoplectrus nigricans	Serranidae	BCF
Hypoplectrus puella	Serranidae	BCF
Hypoplectrus unicolor	Serranidae	BCF
Hyporthodus haifensis	Serranidae	BCF
Hyporthodus niphobles	Serranidae	BCF
Hyporthodus niveatus	Serranidae	BCF
Hyporthodus quernus	Serranidae	BCF
Hyporthodus septemfasciatus	Serranidae	BCF
Liopropoma carmabi	Serranidae	BCF
Liopropoma eukrines	Serranidae	BCF
Luzonichthys waitei	Serranidae	BCF
Mycteroperca acutirostris	Serranidae	BCF
Mycteroperca bonaci	Serranidae	BCF
Mycteroperca interstitialis	Serranidae	BCF
Mycteroperca jordani	Serranidae	BCF
Mycteroperca microlepis	Serranidae	BCF
Mycteroperca phenax	Serranidae	BCF
Mycteroperca prionura	Serranidae	BCF
Mycteroperca rosacea	Serranidae	BCF
Mycteroperca rubra	Serranidae	BCF
Mycteroperca tigris	Serranidae	BCF
Mycteroperca venenosa	Serranidae	BCF
Nemanthias carberryi	Serranidae	BCF
Niphon spinosus	Serranidae	BCF
Paranthias colonus	Serranidae	BCF
Paranthias furcifer	Serranidae	BCF
Plectropomus areolatus	Serranidae	BCF
Plectropomus laevis	Serranidae	BCF
Plectropomus leopardus	Serranidae	BCF
Plectropomus maculatus	Serranidae	BCF
Plectropomus oligacanthus	Serranidae	BCF
Pogonoperca punctata	Serranidae	BCF
Pronotogrammus multifasciatus	Serranidae	BCF
Pseudanthias pleurotaenia	Serranidae	BCF
Pseudanthias squamipinnis	Serranidae	BCF
Schultzea beta	Serranidae	BCF
Serranus atricauda	Serranidae	BCF
Serranus cabrilla	Serranidae	BCF
Serranus phoebe	Serranidae	BCF
Serranus tabacarius	Serranidae	BCF
Serranus tigrinus	Serranidae	BCF
Serranus tortugarum	Serranidae	BCF
Tosana niwae	Serranidae	BCF
Variola albimarginata	Serranidae	BCF
Variola louti	Serranidae	BCF
Serrivomer samoensis	Serrivomeridae	BCF
Serrivomer sector	Serrivomeridae	BCF
Stemonidium hypomelas	Serrivomeridae	BCF
Siganus argenteus	Siganidae	BCF
Siganus canaliculatus	Siganidae	BCF
Siganus corallinus	Siganidae	BCF

(continued)

Species	Family	Locomotion Mode
Siganus doliatus	Siganidae	BCF
Siganus fuscescens	Siganidae	BCF
Siganus guttatus	Siganidae	BCF
Siganus javus	Siganidae	BCF
Siganus lineatus	Siganidae	BCF
Siganus luridus	Siganidae	BCF
Siganus puellus	Siganidae	BCF
Siganus punctatissimus	Siganidae	BCF
Siganus punctatus	Siganidae	BCF
Siganus randalli	Siganidae	BCF
Siganus rivulatus	Siganidae	BCF
Siganus spinus	Siganidae	BCF
Siganus stellatus	Siganidae	BCF
Siganus sutor	Siganidae	BCF
Siganus unimaculatus	Siganidae	BCF
Siganus uspi	Siganidae	BCF
Siganus vermiculatus	Siganidae	BCF
Siganus virgatus	Siganidae	BCF
Siganus vulpinus	Siganidae	BCF
Sillaginodes punctatus	Sillaginidae	BCF
Sillaginopodys chondropus	Sillaginidae	BCF
Sillago aeolus	Sillaginidae	BCF
Sillago analis	Sillaginidae	BCF
Sillago asiatica	Sillaginidae	BCF
Sillago bassensis	Sillaginidae	BCF
Sillago ciliata	Sillaginidae	BCF
Sillago japonica	Sillaginidae	BCF
Sillago parvisquamis	Sillaginidae	BCF
Sillago robusta	Sillaginidae	BCF
Sillago sihama	Sillaginidae	BCF
Acanthopagrus australis	Sparidae	BCF
Acanthopagrus bifasciatus	Sparidae	BCF
Acanthopagrus chinshira	Sparidae	BCF
Acanthopagrus pacificus	Sparidae	BCF
Acanthopagrus schlegelii schlegelii	Sparidae	BCF
Archosargus probatocephalus	Sparidae	BCF
Argyrops bleekeri	Sparidae	BCF
Argyrops spinifer	Sparidae	BCF
Argyrozona argyrozona	Sparidae	BCF
Boops boops	Sparidae	BCF
Calamus brachysomus	Sparidae	BCF
Calamus calamus	Sparidae	BCF
Calamus nodosus	Sparidae	BCF
Calamus penna	Sparidae	BCF
Cheimerius nufar	Sparidae	BCF
Chrysoblephus lophus	Sparidae	BCF
Dentex angolensis	Sparidae	BCF
Dentex canariensis	Sparidae	BCF
Dentex dentex	Sparidae	BCF
Dentex gibbosus	Sparidae	BCF
Dentex macrophthalmus	Sparidae	BCF

(continued)

Species	Family	Locomotion Mode
Dentex maroccanus	Sparidae	BCF
Diplodus annularis	Sparidae	BCF
Diplodus argenteus argenteus	Sparidae	BCF
Diplodus bermudensis	Sparidae	BCF
Diplodus cervinus cervinus	Sparidae	BCF
Diplodus fasciatus	Sparidae	BCF
Diplodus holbrookii	Sparidae	BCF
Diplodus noct	Sparidae	BCF
Diplodus puntazzo	Sparidae	BCF
Diplodus sargus cadenati	Sparidae	BCF
Diplodus sargus sargus	Sparidae	BCF
Diplodus vulgaris	Sparidae	BCF
Evynnis tumifrons	Sparidae	BCF
Lithognathus mormyrus	Sparidae	BCF
Oblada melanura	Sparidae	BCF
Pachymetopon aeneum	Sparidae	BCF
Pagellus acarne	Sparidae	BCF
Pagellus bellottii	Sparidae	BCF
Pagellus bogaraveo	Sparidae	BCF
Pagellus erythrinus	Sparidae	BCF
Pagrus auriga	Sparidae	BCF
Pagrus caeruleostictus	Sparidae	BCF
Pagrus pagrus	Sparidae	BCF
Polysteganus praeorbitalis	Sparidae	BCF
Porcostoma dentata	Sparidae	BCF
Pterogymnus laniarius	Sparidae	BCF
Rhabdosargus holubi	Sparidae	BCF
Rhabdosargus sarba	Sparidae	BCF
Sarpa salpa	Sparidae	BCF
Sparodon durbanensis	Sparidae	BCF
Sparus aurata	Sparidae	BCF
Spondyliosoma cantharus	Sparidae	BCF
Stenotomus chrysops	Sparidae	BCF
Sphyraena argentea	Sphyraenidae	BCF
Sphyraena barracuda	Sphyraenidae	BCF
Sphyraena borealis	Sphyraenidae	BCF
Sphyraena flavicauda	Sphyraenidae	BCF
Sphyraena forsteri	Sphyraenidae	BCF
Sphyraena guachancho	Sphyraenidae	BCF
Sphyraena idiastes	Sphyraenidae	BCF
Sphyraena jello	Sphyraenidae	BCF
Sphyraena obtusata	Sphyraenidae	BCF
Sphyraena picudilla	Sphyraenidae	BCF
Sphyraena putnamae	Sphyraenidae	BCF
Sphyraena sphyraena	Sphyraenidae	BCF
Sphyraena viridensis	Sphyraenidae	BCF
Acanthochaenus luetkenii	Stephanoberycidae	BCF
Argyropelecus aculeatus	Sternoptychidae	BCF
Argyropelecus affinis	Sternoptychidae	BCF
Argyropelecus gigas	Sternoptychidae	BCF
Argyropelecus hemigymnus	Sternoptychidae	BCF

(continued)

Species	Family	Locomotion Mode
Argyropelecus lychnus	Sternoptychidae	BCF
Argyropelecus olfersii	Sternoptychidae	BCF
Argyropelecus sladeni	Sternoptychidae	BCF
Danaphos oculatus	Sternoptychidae	BCF
Maurolicus japonicus	Sternoptychidae	BCF
Maurolicus muelleri	Sternoptychidae	BCF
Maurolicus weitzmani	Sternoptychidae	BCF
Polyipnus asteroides	Sternoptychidae	BCF
Polyipnus clarus	Sternoptychidae	BCF
Polyipnus indicus	Sternoptychidae	BCF
Sternoptyx diaphana	Sternoptychidae	BCF
Sternoptyx pseudobscura	Sternoptychidae	BCF
Aristostomias polydactylus	Stomiidae	BCF
Aristostomias scintillans	Stomiidae	BCF
Aristostomias tittmanni	Stomiidae	BCF
Astronesthes gemmifer	Stomiidae	BCF
Astronesthes macropogon	Stomiidae	BCF
Astronesthes similus	Stomiidae	BCF
Bathophilus pawneei	Stomiidae	BCF
Bathophilus vaillanti	Stomiidae	BCF
Borostomias antarcticus	Stomiidae	BCF
Borostomias elucens	Stomiidae	BCF
Borostomias panamensis	Stomiidae	BCF
Chauliodus danae	Stomiidae	BCF
Chauliodus macouni	Stomiidae	BCF
Chauliodus sloani	Stomiidae	BCF
Echiostoma barbatum	Stomiidae	BCF
Eustomias acinosus	Stomiidae	BCF
Eustomias filifer	Stomiidae	BCF
Eustomias furcifer	Stomiidae	BCF
Eustomias jimcraddocki	Stomiidae	BCF
Eustomias polyaster	Stomiidae	BCF
Flagellostomias boureei	Stomiidae	BCF
Grammatostomias flagellibarba	Stomiidae	BCF
Heterophotus ophistoma	Stomiidae	BCF
Idiacanthus antrostomus	Stomiidae	BCF
Idiacanthus atlanticus	Stomiidae	BCF
Idiacanthus fasciola	Stomiidae	BCF
Leptostomias gladiator	Stomiidae	BCF
Malacosteus niger	Stomiidae	BCF
Melanostomias margaritifer	Stomiidae	BCF
Neonesthes capensis	Stomiidae	BCF
Odontostomias micropogon	Stomiidae	BCF
Pachystomias microdon	Stomiidae	BCF
Photonectes braueri	Stomiidae	BCF
Photonectes dinema	Stomiidae	BCF
Photonectes margarita	Stomiidae	BCF
Rhadinesthes decimus	Stomiidae	BCF
Stomias affinis	Stomiidae	BCF
Stomias atriventer	Stomiidae	BCF
Stomias boa boa	Stomiidae	BCF

(continued)

Species	Family	Locomotion Mode
Stomias gracilis	Stomiidae	BCF
Tactostoma macropus	Stomiidae	BCF
Thysanactis dentex	Stomiidae	BCF
Trigonolampa miriceps	Stomiidae	BCF
Pampus argenteus	Stromateidae	BCF
Peprilus burti	Stromateidae	BCF
Peprilus paru	Stromateidae	BCF
Peprilus simillimus	Stromateidae	BCF
Stromateus brasiliensis	Stromateidae	BCF
Stromateus fiatola	Stromateidae	BCF
Stylephorus chordatus	Stylephoridae	MPF
Symphysanodon berryi	Symphysanodontidae	BCF
Symphysanodon octoactinus	Symphysanodontidae	BCF
Kaupus costatus	Syngnathidae	MPF
Syngnathus pelagicus	Syngnathidae	MPF
Harpadon microchir	Synodontidae	BCF
Pelates quadrilineatus	Terapontidae	BCF
Pelsartia humeralis	Terapontidae	BCF
Tetragonurus cuvieri	Tetragonuridae	BCF
Arothron hispidus	Tetraodontidae	MPF
Arothron immaculatus	Tetraodontidae	MPF
Arothron manilensis	Tetraodontidae	MPF
Arothron mappa	Tetraodontidae	MPF
Arothron nigropunctatus	Tetraodontidae	MPF
Arothron stellatus	Tetraodontidae	MPF
Canthigaster amboinensis	Tetraodontidae	MPF
Canthigaster bennetti	Tetraodontidae	MPF
Canthigaster compressa	Tetraodontidae	MPF
Canthigaster coronata	Tetraodontidae	MPF
Canthigaster figueiredoi	Tetraodontidae	MPF
Canthigaster jactator	Tetraodontidae	MPF
Canthigaster janthinoptera	Tetraodontidae	MPF
Canthigaster leoparda	Tetraodontidae	MPF
Canthigaster natalensis	Tetraodontidae	MPF
Canthigaster papua	Tetraodontidae	MPF
Canthigaster punctatissima	Tetraodontidae	MPF
Canthigaster rivulata	Tetraodontidae	MPF
Canthigaster rostrata	Tetraodontidae	MPF
Canthigaster solandri	Tetraodontidae	MPF
Canthigaster valentini	Tetraodontidae	MPF
Lagocephalus gloveri	Tetraodontidae	MPF
Lagocephalus guentheri	Tetraodontidae	MPF
Lagocephalus inermis	Tetraodontidae	MPF
Lagocephalus laevigatus	Tetraodontidae	MPF
Lagocephalus lagocephalus lagocephalus	Tetraodontidae	MPF
Lagocephalus lunaris	Tetraodontidae	MPF
Lagocephalus sceleratus	Tetraodontidae	MPF
Lagocephalus wheeleri	Tetraodontidae	MPF
Marilyna darwinii	Tetraodontidae	MPF
Sphoeroides annulatus	Tetraodontidae	MPF
Sphoeroides dorsalis	Tetraodontidae	MPF

(continued)

Species	Family	Locomotion Mode
Sphoeroides greeleyi	Tetraodontidae	MPF
Sphoeroides lobatus	Tetraodontidae	MPF
Sphoeroides maculatus	Tetraodontidae	MPF
Sphoeroides nephelus	Tetraodontidae	MPF
Sphoeroides pachygaster	Tetraodontidae	MPF
Sphoeroides spengleri	Tetraodontidae	MPF
Sphoeroides testudineus	Tetraodontidae	MPF
Takifugu bimaculatus	Tetraodontidae	MPF
Takifugu niphobles	Tetraodontidae	MPF
Takifugu oblongus	Tetraodontidae	MPF
Takifugu pardalis	Tetraodontidae	MPF
Takifugu porphyreus	Tetraodontidae	MPF
Takifugu pseudommus	Tetraodontidae	MPF
Takifugu stictonotus	Tetraodontidae	MPF
Takifugu vermicularis	Tetraodontidae	MPF
Takifugu xanthopterus	Tetraodontidae	MPF
Tetractenos hamiltoni	Tetraodontidae	MPF
Torquigener flavimaculosus	Tetraodontidae	MPF
Torquigener hypselogeneion	Tetraodontidae	MPF
Torquigener pleurogramma	Tetraodontidae	MPF
Hoplostethus atlanticus	Trachichthyidae	BCF
Hoplostethus cadenati	Trachichthyidae	BCF
Hoplostethus crassispinus	Trachichthyidae	BCF
Hoplostethus japonicus	Trachichthyidae	BCF
Hoplostethus mediterraneus mediterraneus	Trachichthyidae	BCF
Hoplostethus melanopus	Trachichthyidae	BCF
Hoplostethus occidentalis	Trachichthyidae	BCF
Desmodema polystictum	Trachipteridae	MPF
Trachipterus altivelis	Trachipteridae	MPF
Trachipterus trachypterus	Trachipteridae	MPF
Zu cristatus	Trachipteridae	MPF
Pseudotriacanthus strigilifer	Triacanthidae	BCF
Triacanthus biaculeatus	Triacanthidae	BCF
Trixiphichthys weberi	Triacanthidae	BCF
Tydemania navigatoris	Triacanthodidae	MPF
Aphanopus carbo	Trichiuridae	BCF
Aphanopus intermedius	Trichiuridae	BCF
Benthodesmus elongatus	Trichiuridae	BCF
Benthodesmus simonyi	Trichiuridae	BCF
Evoxymetopon taeniatus	Trichiuridae	BCF
Lepidopus altifrons	Trichiuridae	BCF
Lepidopus caudatus	Trichiuridae	BCF
Lepturacanthus savala	Trichiuridae	BCF
Trichiurus lepturus	Trichiuridae	BCF
Velifer hypselopterus	Veliferidae	BCF
Xiphias gladius	Xiphiidae	BCF
Zanclus cornutus	Zanclidae	MPF
Zenopsis conchifer	Zeidae	MPF
Zenopsis nebulosa	Zeidae	MPF
Zeus capensis	Zeidae	MPF
Zeus faber	Zeidae	MPF
